# Subcutaneous efgartigimod PH20 in generalized myasthenia gravis: A phase 3 randomized noninferiority study (ADAPT-SC) and interim analyses of a long-term open-label extension study (ADAPT-SC+)

**DOI:** 10.1016/j.neurot.2024.e00378

**Published:** 2024-09-02

**Authors:** James F. Howard, Tuan Vu, George Li, Denis Korobko, Marek Smilowski, Li Liu, Fien Gistelinck, Sophie Steeland, Jan Noukens, Benjamin Van Hoorick, Jana Podhorna, Filip Borgions, Yuebing Li, Kimiaki Utsugisawa, Heinz Wiendl, Jan L. De Bleecker, Renato Mantegazza, the ADAPT-SC and ADAPT-SC+ Study Groups

**Affiliations:** a Department of Neurology, The University of North Carolina at Chapel Hill, Chapel Hill, NC, USA; b Department of Neurology, University of South Florida Morsani College of Medicine, Tampa, FL, USA; c Medsol Clinical Research Center, Port Charlotte, FL, USA; d Regional Centre for Multiple Sclerosis and Other Autoimmune System Diseases of the Nervous System, State Novosibirsk Regional Clinical Hospital, Novosibirsk State Medical University, Novosibirsk, Russia; e Department of Hematology and Bone Marrow Transplantation, Medical University of Silesia, Katowice, Poland; f argenx, Ghent, Belgium; g Curare Consulting, Liempde, the Netherlands; h Neuromuscular Center, Cleveland Clinic, Cleveland, OH, USA; i Department of Neurology, Hanamaki General Hospital, Hanamaki, Japan; j Department of Neurology, University of Münster, Münster, Germany; k Department of Neurology and Neuromuscular Reference Center, Ghent University Hospital, Ghent, Belgium; l Department of Neuroimmunology and Neuromuscular Diseases, Fondazione Istituto Neurologico Carlo Besta, Milan, Italy

**Keywords:** Efgartigimod, Myasthenia gravis, FcRn, IgG recycling, Neonatal Fc receptor antagonist

## Abstract

ADAPT-SC (NCT04735432) was designed to evaluate noninferiority of subcutaneous (SC) efgartigimod PH20 to intravenous (IV) efgartigimod in participants with generalized myasthenia gravis (gMG). ADAPT-SC+ (NCT04818671) is an open-label extension study designed to assess long-term safety, tolerability, and efficacy of efgartigimod PH20 SC. Adult participants in ADAPT-SC were randomly assigned to receive a treatment cycle of 4 once-weekly administrations of efgartigimod PH20 SC 1000 ​mg or efgartigimod IV 10 ​mg/kg, followed by 7 weeks of follow-up. Primary endpoint was percentage change from baseline in total immunoglobulin G (IgG) level at week 4 (1 week after the fourth administration). Secondary efficacy endpoints assessed number and percentage of Myasthenia Gravis Activities of Daily Living (MG-ADL) and Quantitative Myasthenia Gravis (QMG) responders and mean change from baseline in total score for each measure. The primary endpoint was met, demonstrating noninferiority in total IgG reduction between efgartigimod PH20 SC 1000 ​mg and efgartigimod IV 10 ​mg/kg. Clinically meaningful improvements were seen as early as 1 week following the first administration in both treatment arms, with maximal improvements at week 4. Continued treatment cycles of efgartigimod PH20 SC in ADAPT-SC+ have demonstrated long-term safety and consistent improvements in MG-ADL total score. Findings from ADAPT-SC and ADAPT-SC+ demonstrate similar safety and efficacy as observed in the placebo-controlled ADAPT study. Collectively, these findings support noninferiority between efgartigimod PH20 SC 1000 ​mg and efgartigimod IV 10 ​mg/kg, as well as long-term safety, tolerability, and efficacy of efgartigimod PH20 SC for treatment of a broad population of patients with gMG.

## Introduction

Myasthenia gravis (MG) is a rare chronic neuromuscular autoimmune disease caused by pathogenic immunoglobulin G (IgG) autoantibodies binding to components of the neuromuscular junction, which impairs neuromuscular transmission [[1], [2], [3]]. Generalized MG (gMG) is characterized by debilitating exertional muscle fatigue and weakness, resulting in difficulties with mobility, speech and swallowing, ocular motility, and respiration [4,5].

Efgartigimod is a human IgG1 Fc fragment, which blocks the neonatal Fc receptor (FcRn) [6]. It is a natural ligand of FcRn, engineered for increased binding affinity to FcRn while retaining characteristic pH-dependent binding in endosomal conditions to outcompete endogenous IgG [6,7]. Efgartigimod prevents recycling of IgG without impacting its production, selectively reducing all IgG subtypes (ie, IgG1, IgG2, IgG3, IgG4), including pathogenic IgG autoantibodies, without affecting other immunoglobulins (ie, IgM, IgA, IgE, IgD) [6]. FcRn also has a critical role in albumin recycling and homeostasis, and decreases in albumin concentration have been shown with some FcRn-targeting monoclonal antibodies [8,9]. In contrast, efgartigimod has been shown neither to reduce serum albumin nor to increase cholesterol levels [6,10,11]. Functions of FcRn, including IgG and albumin recycling as well as antigen presentation, have been previously described [12].

The efgartigimod 10-mg/kg formulation for intravenous (IV) administration (efgartigimod IV) was the first FcRn antagonist approved to treat patients with gMG in multiple regions, including the US, Japan, Europe, Canada, and China [[13], [14], [15], [16], [17]]. The pivotal phase 3 ADAPT study [18] demonstrated the clinical efficacy, safety, and tolerability of efgartigimod IV [10]. In ADAPT, efgartigimod was administered in treatment cycles of 4 once-weekly infusions, with frequency of cycle administration individualized, based on clinical evaluation. Treatment with efgartigimod resulted in consistent and repeatable reductions in total IgG and acetylcholine receptor (AChR) autoantibody (AChR-Ab) levels, and these reductions correlated with clinical improvement as measured by Myasthenia Gravis Activities of Daily Living (MG-ADL) and Quantitative Myasthenia Gravis (QMG) total scores [10]. Additionally, results from ADAPT+ (phase 3 open-label extension (OLE) study of efgartigimod IV in gMG) demonstrated long-term safety and efficacy similar to those seen in ADAPT [19].

To allow subcutaneous (SC) administration, a novel formulation was developed that contains a higher concentration of efgartigimod, as well as recombinant human hyaluronidase PH20 (rHuPH20). rHuPH20 is an enzyme that locally degrades hyaluronan in the SC space, temporarily reducing the barrier to bulk fluid flow, thereby facilitating more-rapid administration of larger volumes [20]. Based on pharmacodynamic (PD) modeling of data from a phase 1 study [21] that investigated various SC doses, the 1000 ​mg efgartigimod PH20 SC injection was estimated to provide similar reduction in total IgG level as the efgartigimod 10 ​mg/kg IV infusion, thus, providing a similar level of clinical efficacy [22]. Efgartigimod PH20 SC injection (5.56 ​mL) typically takes 30–90 ​s, and patients and caregivers can be trained on its administration, allowing the potential for self- (or caregiver) administration.

The efgartigimod IV formulation was approved in the US, EU, China, and Canada for adult patients with acetylcholine receptor antibody–positive (AChR-Ab+) gMG and in Japan regardless of antibody status [[13], [14], [15], [16], [17]]. The efgartigimod PH20 SC formulation was recently approved by the US Food and Drug Administration (2023) for adult patients with AChR-Ab+ gMG [23].

The primary objective of the ADAPT-SC study [24] was to demonstrate noninferiority (NI) of 4 once-weekly administrations of efgartigimod PH20 SC 1000 ​mg to efgartigimod IV 10 ​mg/kg in participants with gMG, as assessed by percentage change from baseline in total IgG at week 4 (ie, 7 days after the fourth SC or IV administration of the treatment cycle). The prespecified NI margin was 10%. An OLE study, ADAPT-SC+ [25] was designed to further assess the long-term safety, tolerability, pharmacodynamics, and efficacy of efgartigimod PH20 SC.

## Methods

### Study designs

ADAPT-SC was a phase 3, randomized, open-label, parallel-group, multicenter clinical trial conducted between February 5, 2021, and December 13, 2021, at 43 sites worldwide. ADAPT-SC was conducted according to the International Conference on Harmonisation of Good Clinical Practice, the principles of the Declaration of Helsinki, and applicable local ethical and legal requirements. Independent ethics committees and institutional review boards provided written approval for the study protocol and all amendments, and an independent data safety monitoring board periodically reviewed and evaluated the accumulated study data for participant safety, study conduct, and study progress. All participants provided written informed consent. Participants who completed ADAPT-SC had the option of entering the OLE ADAPT-SC+ trial to receive additional treatment cycles of efgartigimod PH20 SC 1000 ​mg. ADAPT-SC+ is an ongoing, phase 3, multicenter, open-label, rollover study. This manuscript discusses the complete dataset from ADAPT-SC (1 treatment cycle) and interim results from ADAPT-SC+ (up to 6 treatment cycles of safety data and descriptive summaries on 3 treatment cycles of efficacy/PD data; cutoff date in January 2022 for PD and March 2022 for safety and efficacy).

**Fig. 1:**
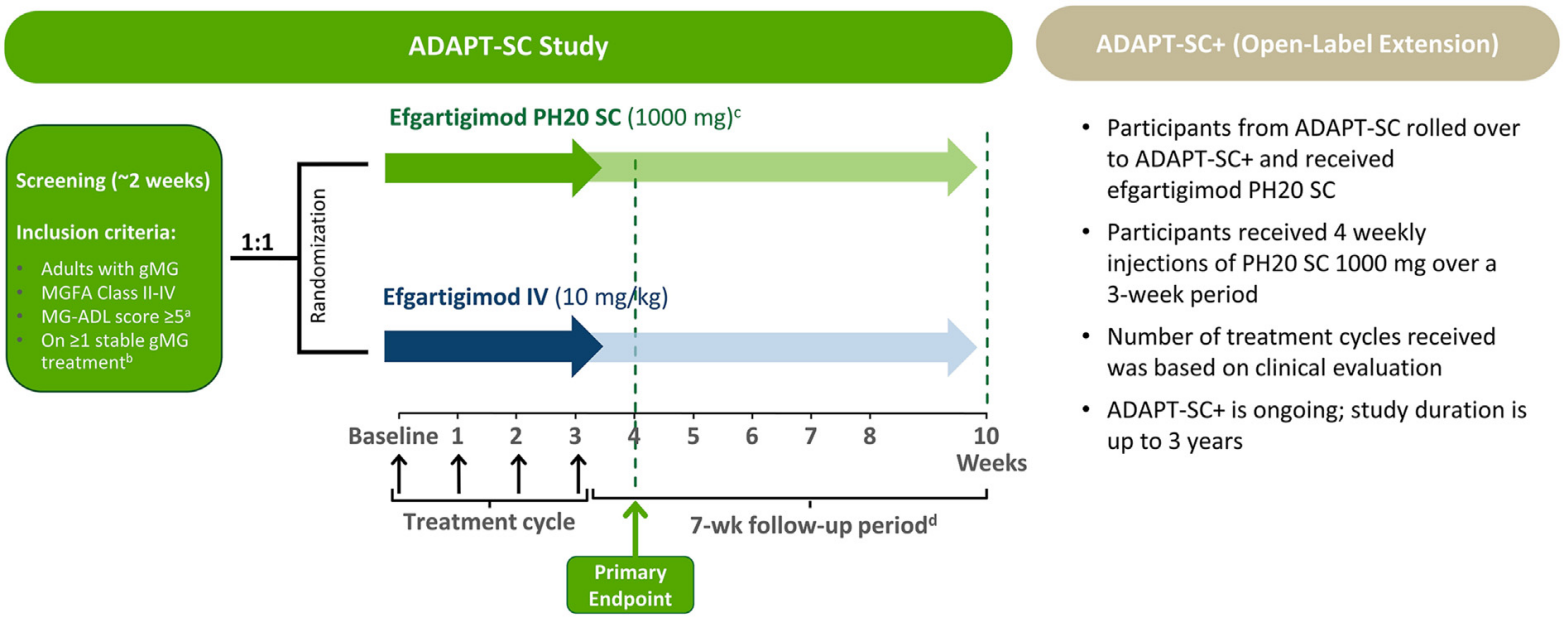
**Study Design Schema for ADAPT-SC and ADAPT-SC+**. AChE, acetylcholinesterase; gMG, generalized myasthenia gravis; IgG, immunoglobulin G; IV, intravenous; IVIg, intravenous immunoglobulin; MG, myasthenia gravis; MG-ADL, Myasthenia Gravis Activities of Daily Living; MGFA, Myasthenia Gravis Foundation of America; NSIST, nonsteroidal immunosuppressive therapy; OLE, open-label extension; PLEX, plasma exchange; rHuPH20, recombinant human hyaluronidase PH20; SC, subcutaneous. Fig. 1 footnote: The ADAPT-SC study evaluated whether the pharmacodynamic effect of efgartigimod PH20 SC 1000 ​mg was noninferior to that of efgartigimod IV 10 ​mg/kg in terms of percentage change from baseline in total IgG level at week 4, using a noninferiority margin of 10%. All participants received 1 treatment cycle (1 injection or infusion per week for 4 weeks, denoted by black arrows) concomitant with their current gMG therapy. The primary endpoint of the ADAPT-SC study was the percentage change from baseline in total IgG levels at week 4 (ie, 1 week after the fourth IV or SC administration). After a participant completed the ADAPT-SC study, the participant was allowed, if eligibility criteria were met, to roll over into ADAPT-SC+ to receive efgartigimod PH20 SC 1000 ​mg. Participants in ADAPT+ (efgartigimod IV OLE) also had the option to roll over into ADAPT-SC+. ^a^ >50% due to nonocular symptoms. ^b^ AChE inhibitors, steroids, and/or NSISTs. ^c^ Efgartigimod PH20 SC 1000 ​mg coformulated with 2000 ​U/mL rHuPH20. ^d^ Participants did not receive treatment, except rescue therapy (corticosteroids, IVIg, and PLEX) and permitted concomitant stable MG therapy, in the 7-week follow-up period.

ADAPT-SC was a 10-week study (detailed in Fig. 1). After screening, participants were randomly assigned 1:1 to receive 1 treatment cycle (4 once-weekly administrations of either efgartigimod PH20 SC 1000 ​mg or efgartigimod IV 10 ​mg/kg), followed by 7 weeks of post-treatment follow-up. Concomitant MG treatments remained at a stable dose throughout the study. Efgartigimod PH20 SC 1000 ​mg injections were administered on site by the study staff; participants (and/or caregivers) who underwent injection training and were determined to be capable were permitted to administer, under supervision of site staff. Efgartigimod IV 10 ​mg/kg was administered as 1-h infusions by site staff. For participants weighing >120 ​kg, the total maximum IV dose was 1200 ​mg. Participants who completed ADAPT-SC had the option of entering ADAPT-SC+ to receive additional treatment cycles of efgartigimod PH20 SC 1000 ​mg. Participants who were in the long-term OLE study of the IV formulation of efgartigimod (ADAPT+) [26] were also able to roll over into the ADAPT-SC+ study if they had completed ≥1 year in that study and received their last dose of efgartigimod IV ​≥30 days prior to entry into ADAPT-SC+.

Study duration of ADAPT-SC+ is up to 3 years. Efgartigimod PH20 SC 1000 ​mg is administered in treatment cycles consisting of 4 once-weekly administrations. Additional cycles are administered based on clinical evaluation.

### Participants

ADAPT-SC enrolled participants aged ≥18 years with gMG, who were either AChR-Ab+ or AChR-Ab negative (AChR-Ab–). MuSK and LRP4 antibodies were not screened for in ADAPT-SC or ADAPT-SC+ so data for these subgroups are not available. Diagnosis of gMG was supported by a history of abnormal neuromuscular transmission demonstrated on electrophysiologic testing, history of positive edrophonium test, or improvement following treatment with acetylcholinesterase (AChE) inhibitors. Participants were required to be Myasthenia Gravis Foundation of America (MGFA) Disease Class II through IV, with an MG-ADL total score of ≥5 points (with >50% of the total score attributed to nonocular symptoms) and to be receiving a stable dose of ≥1 gMG treatment (AChE inhibitors, corticosteroids, and/or nonsteroidal immunosuppressive therapies).

A detailed listing of inclusion and exclusion criteria for ADAPT-SC and ADAPT-SC+ is in Supplemental Table 1.

### Outcomes

The primary endpoint for ADAPT-SC was the percentage change from baseline in total IgG level at week 4 (1 week after the fourth administration), which was used to demonstrate the NI of efgartigimod PH20 SC 1000 ​mg to efgartigimod IV 10 ​mg/kg in participants with gMG. Secondary PD endpoints evaluated percentage change from baseline in total IgG and IgG subtypes with efgartigimod PH20 SC and efgartigimod IV over time in all participants and AChR-Ab levels in AChR-Ab+ participants.

Secondary efficacy endpoints included percentage of MG-ADL and QMG responders and change from baseline in MG-ADL and QMG total scores. “MG-ADL responder” was defined as a participant with a reduction of ≥2 points from baseline for ≥4 consecutive weeks, with onset of score reduction occurring, at the latest, 1 week after the last infusion or injection. “QMG responder” was defined as a participant with a reduction of ≥3 points from baseline for ≥4 consecutive weeks, with onset of score reduction occurring, at the latest, 1 week after the last infusion or injection. Responder definitions mirror the standards for a clinically meaningful improvement (CMI) in MG-ADL and QMG total scores [27,28].

Safety was assessed by incidence and severity of adverse events (AEs; per National Cancer Institute Common Terminology Criteria for Adverse Events) and changes in clinical laboratory values, vital signs, and electrocardiograms. Standardised MedDRA (Medical Dictionary for Regulatory Activities) Queries (SMQ) narrow search was used to identify opportunistic infections. Immunogenicity was evaluated with antidrug antibodies (ADAs) and neutralizing antibodies (NAbs) against efgartigimod and antibodies against rHuPH20.

In ADAPT-SC+, the primary objective is to assess long-term safety and tolerability of efgartigimod PH20 SC 1000 ​mg, with secondary objectives including PD changes and efficacy (using MG-ADL only; QMG is not measured in ADAPT-SC+).

### Statistical analysis

The primary endpoint in ADAPT-SC was assessed using an analysis of covariance model, with treatment arm as a factor and total IgG level at baseline as a covariate. NI evaluation was based on the 2-sided 95% CI of the least squares mean (LSM) difference (efgartigimod PH20 SC vs efgartigimod IV) in percentage change from baseline in total IgG level at week 4, using a NI margin of 10%. Secondary endpoints were summarized with descriptive statistics, by treatment arm and for all participants. Clinical efficacy analyses were performed on the intent-to-treat (ITT) analysis set and PD analyses were performed on the modified intent-to-treat (mITT) analysis set. The ITT analysis set included all randomly assigned participants who were exposed to the investigational medicinal product (IMP); the mITT analysis set included all randomly assigned participants who had a value for total IgG level at baseline and ≥1 postbaseline value. The safety analysis set included all randomly assigned participants who were exposed to the IMP. Subgroup analyses were conducted by AChR-Ab status.

In ADAPT-SC+, the primary and secondary endpoints are summarized using descriptive statistics for the overall population and by AChR-Ab status. Analyses are performed on the safety analysis set, which includes all participants who receive ≥1 dose of efgartigimod PH20 SC in the ADAPT-SC+ study.

## Results

### Participants

**Fig. 2:**
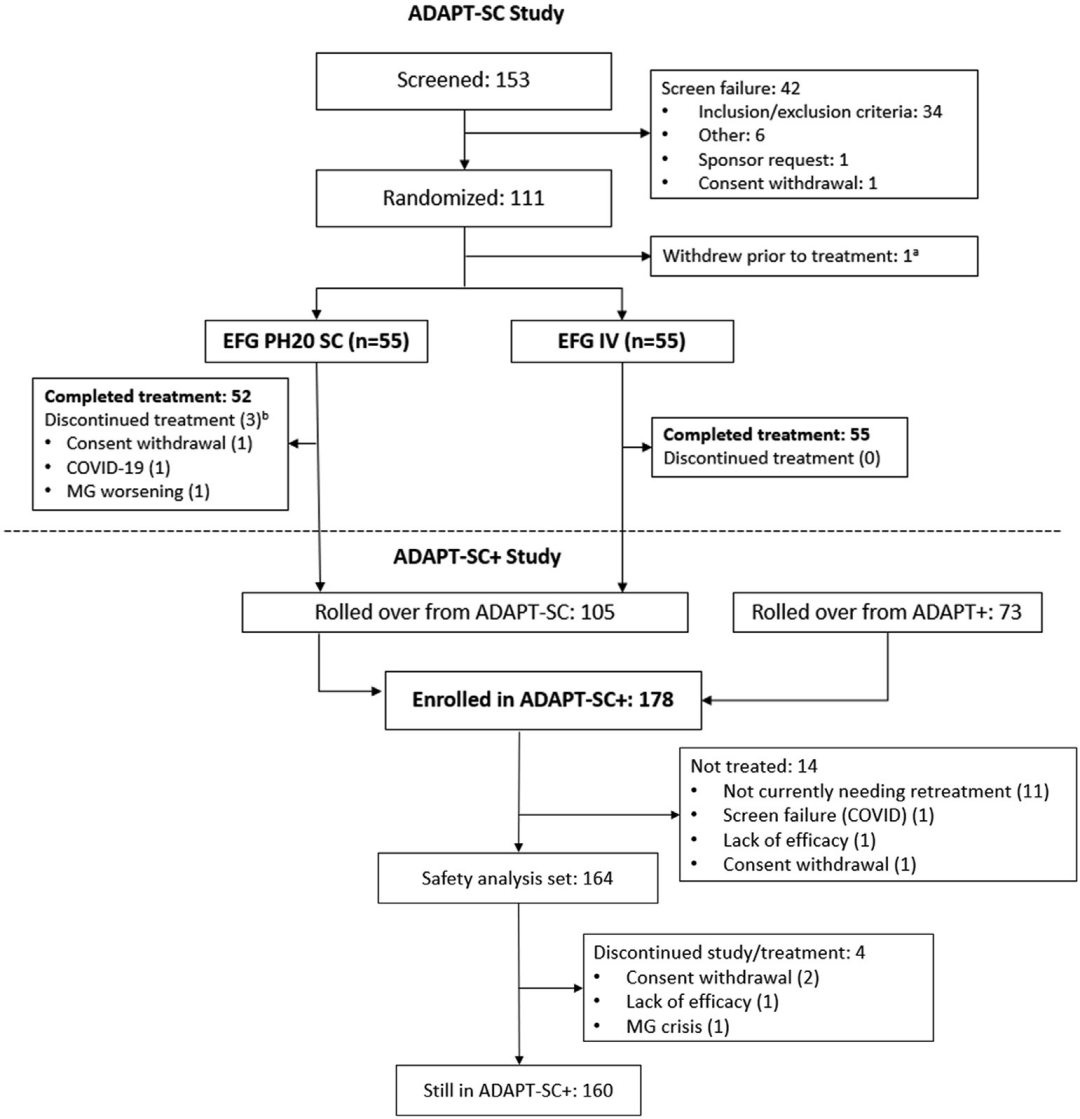
**Participant Disposition (ADAPT-SC and ADAPT-SC+).** AE, adverse event; EFG, efgartigimod; IV, intravenous; MG, myasthenia gravis; SC, subcutaneous. Fig. 2 footnote: Of the 153 participants screened for ADAPT-SC, 111 were enrolled and randomly assigned to receive efgartigimod PH20 SC (n ​= ​55) or efgartigimod IV (n ​= ​55). A total of 110 participants were treated, 107 completed treatment, and 108 completed the study (54 from each treatment arm). In the efgartigimod PH20 SC arm, 3 participants did not complete treatment, and 1 of the 3 also discontinued from the study. In the efgartigimod IV arm, no participant discontinued treatment, but 1 participant discontinued from the study. Of the 178 participants enrolled in ADAPT-SC+, 105 rolled over from the ADAPT-SC study and 73 rolled over from the ADAPT+ study, an open-label extension study of efgartigimod IV. ^a^ 1 participant assigned to the efgartigimod IV arm withdrew due to an AE of pyrexia prior to administration of efgartigimod. ^b^ Treatment discontinuation due to COVID-19 infection occurred on day 3 and MG worsening on day 1.

Of the 153 participants screened for ADAPT-SC, 111 were enrolled (Fig. 2); 55 were randomly assigned to efgartigimod PH20 SC and 55 to efgartigimod IV. One participant assigned to the efgartigimod IV arm withdrew due to an AE of pyrexia prior to administration of efgartigimod. There was no difference in the number of participants in the mITT, ITT, and safety analysis populations (n ​= ​110 for each) for ADAPT-SC. Participant characteristics were representative of a broad gMG population and were well balanced between treatment arms (Table 1). Baseline MG-ADL and QMG scores were comparable between treatment arms and indicated continuing disease burden [29]. Most participants (82.7%) were AChR-Ab+.

**Table 1:** Participant demographics and baseline characteristics of ADAPT-SC and ADAPT-SC+.

	ADAPT-SC (PD Noninferiority Study)				ADAPT-SC+ (Open-Label Extension Study)	
	Overall participants		AChR-Ab–positive participants		Overall participants	AChR-Ab–positive participants
	EFG PH20 SC (n ​= ​55)	EFG IV (n ​= ​55)	EFG PH20 SC (n ​= ​45)	EFG IV (n ​= ​46)	EFG PH20 SC (n ​= ​164)	EFG PH20 SC (n ​= ​134)
Age (years)						
Mean (SD)	50.9 (15.8)	55.8 (15.4)	51.3 (16.3)	57.0 (14.8)	50.7 (15.4)	51.2 (15.9)
Median (min, max)	53.0 (19, 84)	59.0 (24, 83)	53.0 (19, 84)	60.0 (24, 83)	50.0 (19, 84)	53.0 (19, 84)
Sex, n (%)						
Female	31 (56.4)	34 (61.8)	25 (55.6)	26 (56.5)	106 (64.6)	83 (61.9)
Male	24 (43.6)	21 (38.2)	20 (44.4)	20 (43.5)	58 (35.4)	51 (38.1)
Race, n (%)						
Asian (Japanese)	4 (7.3)	4 (7.3)	3 (6.7)	3 (6.5)	14 (8.5)	9 (6.7)
Black or African American	0	0	0	0	2 (1.2)	1 (0.7)
Multiple	1 (1.8)	0	2 (2.2)	0	1 (0.6)	1 (0.7)
White	50 (90.9)	51 (92.7)	52 (91.1)	43 (93.5)	147 (89.6)	123 (91.8)
Weight (kg)						
Mean (SD)	79.8 (21.1)	81.5 (22.2)	77.3 (19.6)	83.9 (22.8)	79.2 (20.6)	79.9 (20.8)
Median (min, max)	78.3 (42.0, 150.2)	78.0 (45.0, 139.3)	76.9 (42.0, 115.4)	81.7 (46.9, 139.3)	77.0 (43.9, 148.8)	78.0 (43.9, 148.8)
Time since gMG diagnosis (years)						
Mean (SD)	6.3 (6.4)	7.7 (8.5)	6.7 (6.7)	7.9 (8.9)	8.9 (8.1)	9.1 (8.6)
Median (min, max)	4.4 (0.5, 33.0)	4.6 (0.1, 40.4)	4.6 (0.6, 33.0)	4.0 (0.1, 40.4)	5.9 (0.4, 48.1)	5.9 (0.4, 48.1)
Previous thymectomy, n (%)						
Yes	16 (29.1)	13 (23.6)	14 (31.1)	12 (26.1)	62 (37.8)	54 (40.3)
MGFA class at screening, n (%)^a^						
Class II	29 (52.7)	22 (40.0)	25 (55.6)	17 (37.0)	65 (39.6)	55 (41.0)
Class III	24 (43.7)	30 (54.5)	19 (42.2)	27 (58.7)	92 (56.1)	74 (55.2)
Class IV	2 (3.6)	3 (5.5)	1 (2.2)	2 (4.3)	7 (4.3)	5 (3.7)
MG-ADL total score at baseline						
Mean (SD)	8.8 (2.6)	8.5 (2.6)	8.6 (2.6)	8.3 (2.5)	7.9 (3.5)	7.6 (3.5)
Median (min, max)	8.0 (5, 16)	8.0 (5, 15)	8.0 (5, 16)	8.0 (5, 15)	8.0 (0, 20)	7.0 (0, 20)
QMG total score at baseline						
Mean (SD)	14.9 (4.4)	15.5 (4.5)	14.4 (4.4)	15.1 (4.3)	NA	NA
Median (min, max)	15.0 (3, 25)	16.0 (7, 27)	14.0 (3, 25)	15.0 (7, 25)	NA	NA
AChR-Ab status, n (%)						
AChR-Ab–positive	45 (81.8)	46 (83.6)	45 (100)	46 (100)	134 (81.7)	134 (100.0)
AChR-Ab–negative	10 (18.2)	9 (16.4)	–	–	30 (18.3)	–
Concomitant MG therapy, n (%)^b^						
Any steroid^c^	40 (72.7)	33 (60.0)	34 (75.6)	29 (63.0)	112 (68.3)	93 (69.4)
Any NSIST^c^	23 (41.8)	25 (45.5)	18 (40.0)	19 (41.3)	84 (51.2)	65 (48.5)
Any AChE inhibitor	48 (87.3)	47 (85.5)	39 (86.7)	39 (84.8)	140 (85.4)	118 (88.1)
Steroid ​+ ​NSIST^c^	19 (34.5)	16 (29.1)	16 (35.6)	13 (28.3)	64 (39.0)	50 (37.3)
AChE inhibitor only	11 (20.0)	12 (21.8)	9 (20.0)	10 (21.7)	30 (18.3)	25 (18.7)

AChE, acetylcholinesterase; AChR-Ab, acetylcholine receptor antibody; EFG, efgartigimod; gMG, generalized myasthenia gravis; IV, intravenous; MG, myasthenia gravis; MG-ADL, Myasthenia Gravis Activities of Daily Living; MGFA, Myasthenia Gravis Foundation of America; NA, not available; NSIST, nonsteroidal immunosuppressive therapy; PD, pharmacodynamics; QMG, Quantitative Myasthenia Gravis; SC, subcutaneous.

a For ADAPT-SC+, this was at screening in the antecedent study, ie, ADAPT-SC or ADAPT.

b For ADAPT-SC+, concomitant medications represent those used during the first year of the study.

c Allowed NSISTs included azathioprine, cyclosporine, cyclophosphamide, methotrexate, mycophenolate mofetil, and tacrolimus. May also include the use of an AChE inhibitor.

ADAPT-SC+ enrolled 178 participants: 105 from ADAPT-SC and 73 from ADAPT+. Of those enrolled, 164 have received ≥1 dose of efgartigimod PH20 SC and 81.7% (134/164) are AChR-Ab+. Fourteen participants were not in need of treatment or were not treated for various reasons (Fig. 2). At the time of data cutoff, most participants (97.6%; 160/164) were ongoing in the study, with 142 proceeded to cycle 2 and 105 to cycle 3; mean duration of follow-up is 169.7 days, corresponding to 72.1 participant-years of exposure.

### Pharmacodynamic and efficacy endpoints

The ADAPT-SC primary endpoint was met. The LSM estimate of percentage change from baseline in total IgG level at week 4 was −66.4% for the efgartigimod PH20 SC arm vs −62.2% for the efgartigimod IV arm (Table 2), giving a LSM difference of −4.2% (95% CI, −7.73 to −0.66). The upper limit of the 2-sided 95% CI (−0.66%) is below the 10% NI margin, indicating the primary endpoint for ADAPT-SC was met. Similar results were observed for the AChR-Ab+ population.

**Table 2:** Percentage Change From Baseline in Total IgG Level at Week 4 of ADAPT-SC (Primary Endpoint, mITT Population).

	EFG PH20 SC			EFG IV			EFG PH20 SC vs EFG IV		
	n	LSM difference	95% CI	n	LSM difference	95% CI	LSM difference	95% CI	*P* value
Overall	50	−66.4	−68.91 to −63.86	52	−62.2	−64.67 to −59.72	−4.2	−7.73 to −0.66	<.0001
AChR-Ab+	41	−66.9	−69.78 to −64.02	43	−62.4	−65.22 to −59.59	−4.5	−8.53 to −0.46	<.0001

AChR-Ab, acetylcholine receptor antibody; EFG, efgartigimod; IgG, immunoglobulin G; IV, intravenous; LSM, least squares mean; mITT, modified intent to treat; SC, subcutaneous.

Table 2 footnote: mITT analysis set included all randomly assigned participants with a value for total IgG level at baseline and at least 1 postbaseline time point.

**Fig. 3:**
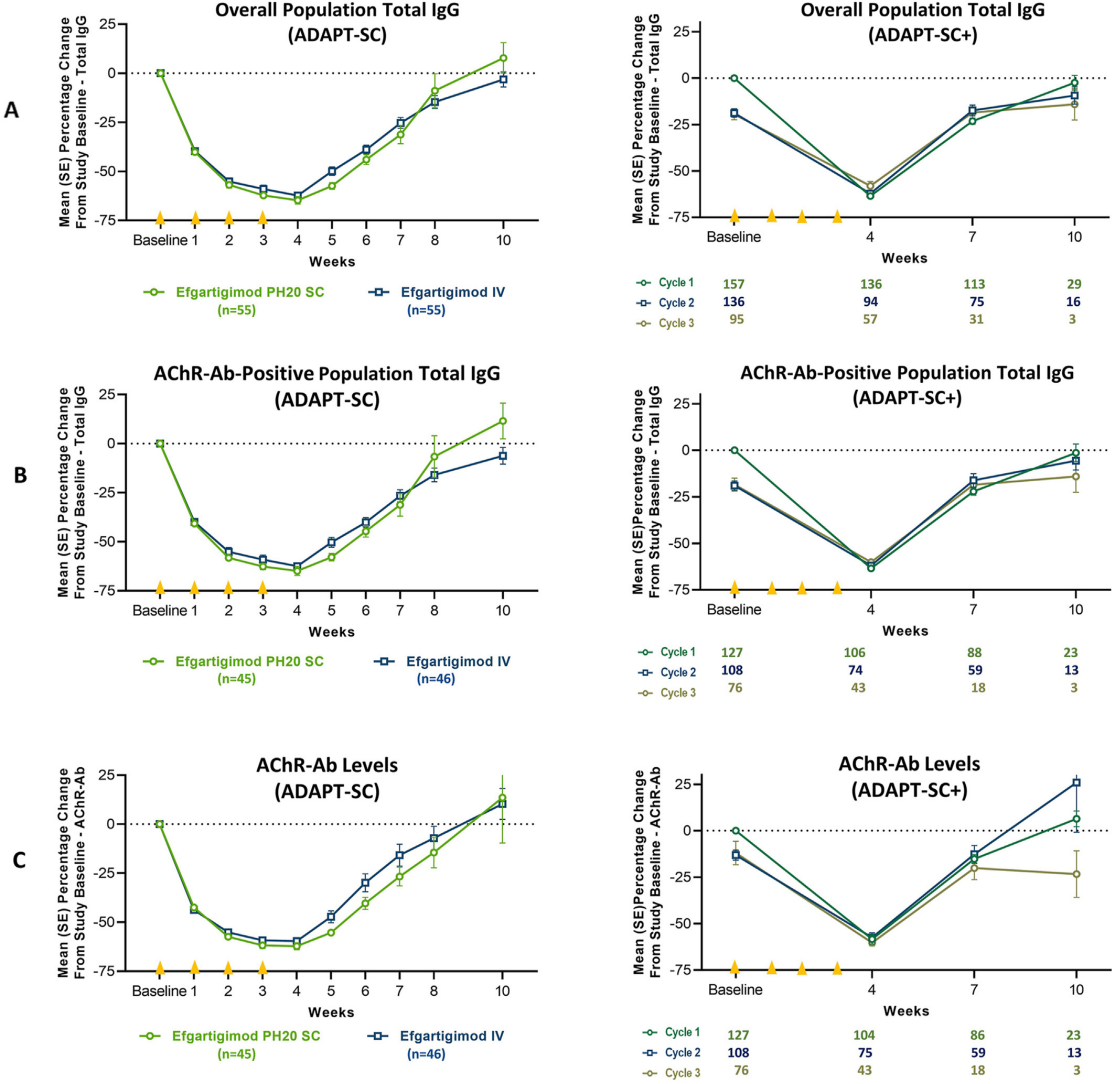
**Mean Percentage Change From Baseline in Total IgG and AChR-Ab Levels—Overall and AChR-Ab+ Populations**^**a**^**—in ADAPT-SC and ADAPT-SC+**. AChR-Ab, acetylcholine receptor antibody; IgG, immunoglobulin G; IMP, investigational medicinal product; IV, intravenous; mITT, modified intent to treat; SC, subcutaneous. Fig. 3 footnote: **A**: Mean percentage change from baseline in total IgG in overall populations in ADAPT-SC and ADAPT-SC+; **B:** Mean percentage change from baseline in total IgG in AChR-Ab–positive subgroups in ADAPT-SC and ADAPT-SC+; **C:** Mean percentage change from baseline in AChR-Ab levels in ADAPT-SC and ADAPT-SC+ (in AChR-Ab–positive participants). The mITT analysis set included all randomly assigned participants with a value for total IgG level at baseline and at least 1 postbaseline time point. Each cycle consisted of 4 once-weekly administrations, occurring at baseline and weeks 1, 2, and 3 (denoted by yellow triangles on the x-axis). ^a^ Data for AChR-Ab–negative participants are in Supplemental Table 2 and Supplemental Fig. 1.

**Fig. S1:**
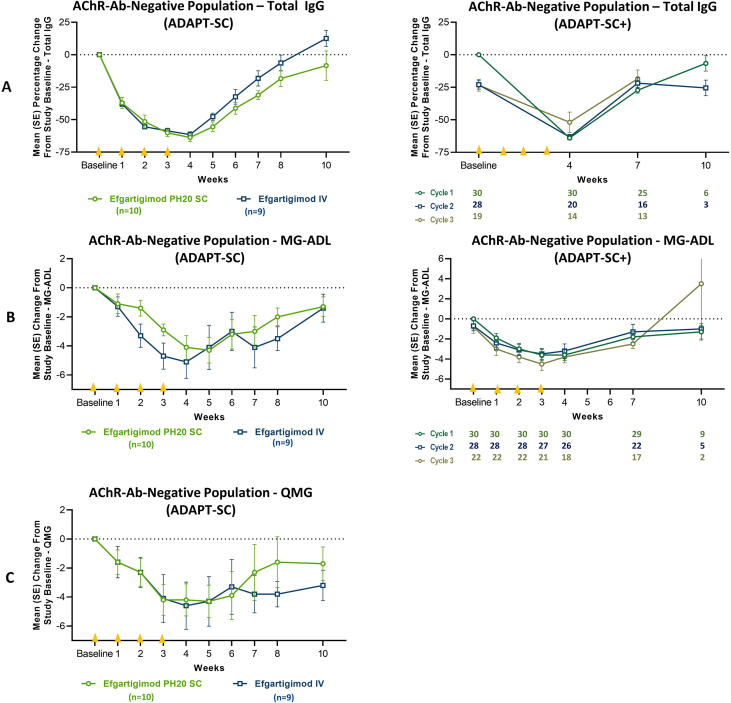
Mean Percentage Change From Baseline in Total IgG (mITT Population) and Mean Change From Baseline in MG-ADL and QMG Total Scores (ITT Population)—AChR-Ab–Negative Population (ADAPT-SC and ADAPT-SC+) AChR-Ab, acetylcholine receptor antibody; IgG, immunoglobulin G; ITT, intent to treat; IV, intravenous; MG-ADL, Myasthenia Gravis Activities of Daily Living; mITT, modified intent to treat; QMG, Quantitative Myasthenia Gravis; SC, subcutaneous. **Supplemental Figure 1 footnote**: **A**: Mean percentage change from study baseline in total IgG in AChR-Ab–negative population in ADAPT-SC (left) and ADAPT-SC+ (right); **B:** mean change from study baseline in MG-ADL total score in AChR-Ab–negative population in ADAPT-SC (left) and ADAPT-SC+ (right); **C**: mean change from study baseline in QMG total score in AChR-Ab–negative population in ADAPT-SC. The ITT analysis set included all randomly assigned participants who were exposed to the IMP. The mITT analysis set included all randomly assigned participants with a value for total IgG level at baseline and at least 1 postbaseline time point. Each cycle consisted of 4 once-weekly administrations, occurring at baseline and weeks 1, 2, and 3 (denoted by yellow triangles on x-axis).

One week after the first administration in ADAPT-SC, mean percentage change in total IgG was −40.1% in the efgartigimod PH20 SC arm and −39.6% in the efgartigimod IV arm (Fig. 3A, Left). Total IgG decreased further after each subsequent administration, with maximal reduction (range, −58.1 to −63.5%) observed at week 4 (ie, 1 week after the fourth administration). Total IgG levels returned to near baseline levels by week 10. Percentage changes in IgG subtypes were consistent with total IgG and similar between treatment arms. Percentage change in total IgG in AChR-Ab+ (Fig. 3B, Left) and AChR-Ab– participants (Supplemental Fig. 1) was similar in both treatment arms and similar to the percentage change in the overall population. In ADAPT-SC+, the percentage reduction from study baseline in total IgG was consistent and repeatable in both the overall and AChR-Ab+ populations, with each cycle showing reductions similar to those seen at equivalent time points in ADAPT-SC (Fig. 3A and B, Right).

AChR-Ab levels were assessed over time in ADAPT-SC and ADAPT-SC+ (Fig. 3C). Reductions in AChR-Ab levels were similar between the efgartigimod PH20 SC and efgartigimod IV arms in ADAPT-SC (Fig. 3C, Left), and the maximum mean reduction in AChR-Ab level was observed at week 4: 62.2% and 59.6% in the efgartigimod PH20 SC and efgartigimod IV arms, respectively. As of data cutoff in ADAPT-SC+, the maximum mean reduction at week 4 ranged from 57.5% to 60.3% across the 3 treatment cycles (Fig. 3C, Right). Changes in AChR-Ab levels paralleled changes seen in total IgG in both studies.

**Fig. 4:**
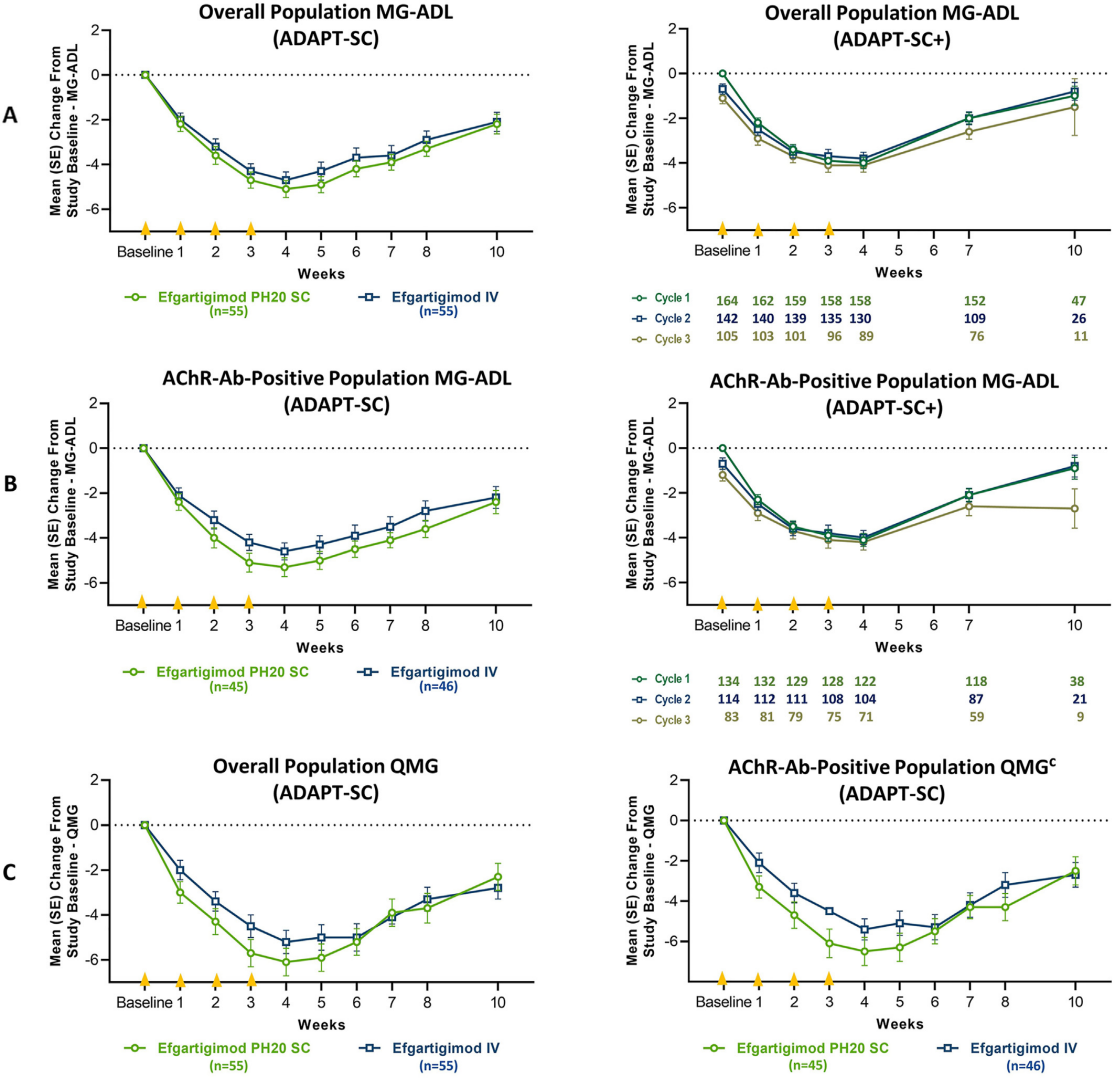
**Mean Change From Baseline in MG-ADL and QMG**^**a**^**Total Scores—Overall and AChR-Ab+ Populations**^**b**^**—in ADAPT-SC and ADAPT-SC+**. AChR-Ab, acetylcholine receptor antibody; IgG, immunoglobulin G; IMP, investigational medicinal product; ITT, intent to treat; IV, intravenous; MG-ADL, Myasthenia Gravis Activities of Daily Living; QMG, Quantitative Myasthenia Gravis; SC, subcutaneous. Fig. 4 footnote: **A**: Mean change from baseline in MG-ADL total score (overall) in ADAPT-SC and ADAPT-SC+; **B**: mean change from baseline in MG-ADL total score (AChR-Ab–positive) in ADAPT-SC and ADAPT-SC+; **C**: mean change from baseline in QMG total score (overall, left and AChR-Ab–positive, right) in ADAPT-SC. The ITT analysis set included all randomly assigned participants who were exposed to the IMP. Each cycle consisted of 4 once-weekly administrations, occurring at baseline and weeks 1, 2, and 3 (denoted by yellow triangles on the x-axis). ^a^ QMG was measured in ADAPT-SC study only, not in ADAPT-SC+. ^b^ Data for AChR-Ab–negative participants are in Supplemental Table 2 and Supplemental Fig. 1. ^c^ Differential scaling of y-axis on AChR-Ab–positive QMG graph to accommodate error bars.

Efficacy of efgartigimod PH20 SC in ADAPT-SC, assessed by MG-ADL and QMG total scores, was similar to efgartigimod IV (Fig. 4). The percentage of MG-ADL responders in the overall population was identical in the efgartigimod PH20 SC and efgartigimod IV arms, and for QMG, there was a similar percentage of responders in the efgartigimod PH20 SC arm as in the efgartigimod IV arm (Table 3). Analysis of AChR-Ab+ participants in the efgartigimod PH20 SC and efgartigimod IV arms demonstrated similar percentages of MG-ADL responders (efgartigimod PH20 SC: 71.1%; efgartigimod IV: 71.7%) and QMG responders (efgartigimod PH20 SC: 68.9%; efgartigimod IV: 53.3%). Clinical improvements were also observed for AChR-Ab− participants, with notable percentages of MG-ADL responders (efgartigimod PH20 SC: 60.0%; efgartigimod IV: 55.6%) and QMG responders (efgartigimod PH20 SC: 50.0%; efgartigimod IV: 44.4%) (Supplemental Table 2).

**Table 3:** Number and percentage of MG-ADL and QMG responders in ADAPT-SC (overall and AChR-Ab+ populations of ITT analysis set)^a^.

	EFG PH20 SC n/N (%)	EFG IV n/N (%)	Difference in response, % (95% CI)
**MG-ADL responders**			
Overall	38/55 (69.1)	38/55 (69.1)	0.0 (−17.3 to 17.3)
AChR-Ab–positive	32/45 (71.1)	33/46 (71.7)	−0.6 (−19.2 to 17.9)
**QMG responders**			
Overall	36/55 (65.5)	28/54 (51.9)	13.6 (−4.7 to 31.9)
AChR-Ab–positive	31/45 (68.9)	24/45 (53.3)	15.6 (−4.3 to 35.4)

AChR-Ab, acetylcholine receptor antibody; EFG, efgartigimod; IMP, investigational medicinal product; ITT, intent to treat; IV, intravenous; MG-ADL, Myasthenia Gravis Activities of Daily Living; n/N, number of participants for whom the observation was reported/number of participants in the analysis set; QMG, Quantitative Myasthenia Gravis; SC, subcutaneous.

Table 3 footnote: ITT analysis set included all randomly assigned participants who were exposed to the IMP. The 95% CI for the difference in the percentage of MG-ADL and QMG responders in the 2 treatment arms was determined based on a two-sample *t*-test using Satterthwaite’s correction. “MG-ADL responder” was defined as a participant with a reduction of ≥2 points from baseline for ≥4 consecutive weeks after onset, with onset of score reduction occurring, at the latest, 1 week after the last infusion or injection. “QMG responder” was defined as a participant with a reduction of ≥3 points from baseline for ≥4 consecutive weeks after onset, with onset of score reduction occurring, at the latest, 1 week after the last infusion or injection.

a Data on AChR-Ab–negative participants are in Supplemental Table 2 and Supplemental Fig. 1.

CMIs were seen as early as 1 week after the first administration in both treatment arms in ADAPT-SC, with maximal improvement in MG-ADL and QMG scores observed at week 4, which aligned with the maximum reduction in total IgG. In the overall population, the mean (SE) change in MG-ADL total score at week 4 was −5.1 (0.38) for the efgartigimod PH20 SC arm and −4.7 (0.37) for the efgartigimod IV arm. Mean (SE) change in QMG total score at week 4 was −6.1 (0.62) for the efgartigimod PH20 SC arm and −5.2 (0.52) for the efgartigimod IV arm (Fig. 4A and C, Left). Similar results in mean (SE) change at week 4 in MG-ADL (efgartigimod PH20 SC: −5.3 [0.42]; efgartigimod IV: −4.6 [0.38]) and QMG (efgartigimod PH20 SC: −6.5 [0.70]; efgartigimod IV: −5.4 [0.53]) scores were seen in AChR-Ab+ participants (Fig. 4B, Left and 4C, Right).

**Fig. 5:**
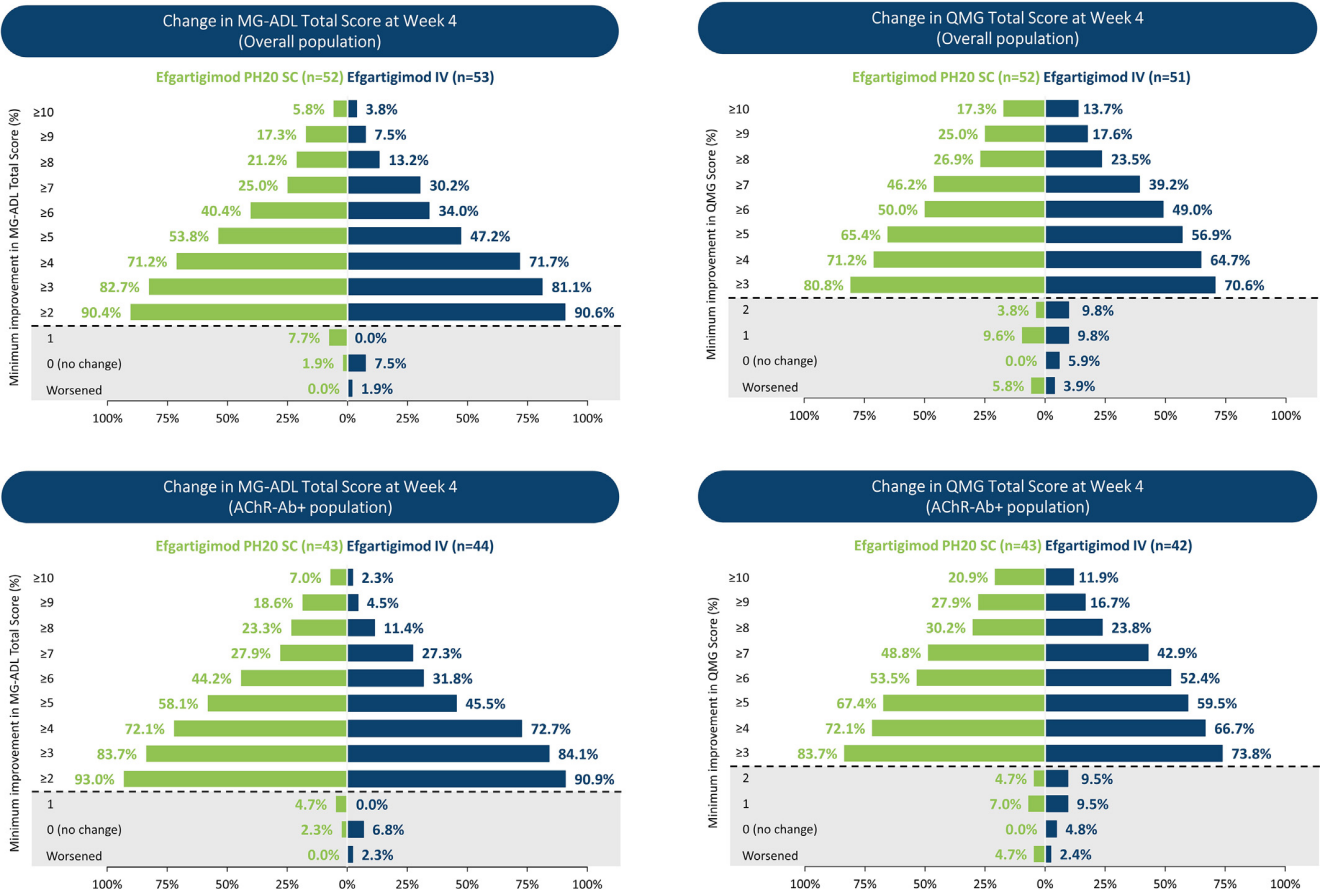
**Proportion of Participants With Increasing MG-ADL or QMG Improvement in ADAPT-SC Study (Overall and AChR-Ab+Populations, Week 4; ITT Population).** AChR-Ab, acetylcholine receptor antibody; AChR-Ab+, acetylcholine receptor antibody–positive; IMP, investigational medicinal product; ITT, intent to treat; IV, intravenous; MG-ADL, Myasthenia Gravis Activities of Daily Living; QMG, Quantitative Myasthenia Gravis; SC, subcutaneous. Fig. 5 footnote: Scores above dashed line indicate clinically meaningful improvement. Values below the dashed lines are actual, not cumulative, percentages. ITT analysis set included all randomly assigned participants who were exposed to the IMP.

In the overall population, 20 of 54 participants (37.0%) in the efgartigimod PH20 SC arm and 21 of 55 (38.2%) in the efgartigimod IV arm achieved minimal symptom expression (MSE; defined as MG-ADL total score of 0 or 1) at any time point during ADAPT-SC. Similar results were seen for the AChR-Ab+ population, with MSE achieved by 20 of 44 (45.5%) in the efgartigimod PH20 SC arm and 19 of 46 (41.3%) in the efgartigimod IV arm at any time point. CMIs were demonstrated at week 4 in the vast majority of participants in the overall population for both treatment arms. The proportion of participants in the efgartigimod PH20 SC and efgartigimod IV arms achieving a ≥2-point improvement in MG-ADL total score was 90.4% and 90.6%, respectively, and a ≥3-point improvement in QMG was 80.8% and 70.6%, respectively (Fig. 5). Similar results were seen at week 4 in the AChR-Ab+ population, with 93.0% and 90.9% of the participants achieving a ≥2-point improvement in MG-ADL total score, as well as 83.7% and 73.8% achieving a ≥3-point improvement in QMG, for efgartigimod PH20 SC and efgartigimod IV treatment arms, respectively. In the overall population, ≥2-point improvement in MG-ADL was maintained at week 10 in 56.5% of efgartigimod PH20 SC participants and in 62.7% of efgartigimod IV participants, with similar results seen in the AChR-Ab+ population (62.2% of participants in the efgartigimod PH20 SC arm and 66.7% of participants in the efgartigimod IV arm). Additionally, in the overall population, participants in the efgartigimod PH20 SC and efgartigimod IV treatment arms demonstrated MG-ADL improvements well beyond CMI thresholds, with 25.0% and 30.2% of participants, respectively, achieving a ≥7-point improvement in MG-ADL at any time during the study (Fig. 5). Similar clinical improvements beyond CMI thresholds were also demonstrated for QMG total score: 26.9% and 23.5% of the participants achieved a ≥8-point improvement at any time during the study in the efgartigimod PH20 SC and IV treatment arms, respectively.

As of data cutoff in ADAPT-SC+, the mean (SE) MG-ADL changes from study baseline at week 4 in the overall population were −4.0 (0.25), −3.8 (0.29), and −4.1 (0.31) in cycles 1 through 3, respectively. Similar results were seen in the AChR-Ab+ population (−4.1 [0.29], −4.0 [0.32], and −4.2 [0.35]) (Fig. 4A and B, Right). As was seen in ADAPT-SC, maximum improvement in MG-ADL total score occurred at week 4, which aligned with the maximum reduction in total IgG. In the overall population, MSE at any time point within a cycle was achieved by 30.2%, 31.2%, and 35.9% of participants in cycles 1 through 3, respectively; similar results were seen in the AChR-Ab+ population (33.3%, 34.5%, and 39.5% in cycles 1–3, respectively). Clinical improvements in AChR-Ab− participants were seen in cycles 1 through 3, with mean (SE) changes from study baseline in MG-ADL at week 4 of −3.6 (0.53), −3.2 (0.71), and −3.8 (0.59), respectively (Supplemental Fig. 1B, Right).

In ADAPT-SC, injections were predominantly administered by site staff; however, participant/caregiver administration, including at-home administration, has been common in ADAPT-SC+: 88% (145/164) of participants in ADAPT-SC+ have been considered to be adequately trained to self-administer efgartigimod PH20 SC, the majority within the first 3 administrations. By treatment cycle 3 of ADAPT-SC+, nearly 60% of all participants have been able to self-administer in their own homes. In cycle 1 of ADAPT-SC+, 71.2% (74/104 participants who received efgartigimod IV in either ADAPT+ [n ​= ​60] or ADAPT-SC [n ​= ​44]) indicated a preference for efgartigimod PH20 SC, while 28.9% (30/104) either preferred efgartigimod IV or had no preference.

### Safety

Efgartigimod PH20 SC and efgartigimod IV were well tolerated in ADAPT-SC; as of data cutoff in ADAPT-SC+, efgartigimod PH20 SC demonstrated long-term safety. In both studies, most AEs were mild to moderate in severity. See Table 4 for an overview of AEs in ADAPT-SC and ADAPT-SC+. No deaths occurred during ADAPT-SC; there were 2 deaths in ADAPT-SC+: 1 due to metastatic renal cancer and 1 due to respiratory failure secondary to COVID-19 infection (participant was not vaccinated against COVID-19). Neither was considered by the investigator to be related to efgartigimod. In the efgartigimod PH20 SC arm of ADAPT-SC, 37 (67.3%) participants had AEs vs 28 (50.9%) participants in the efgartigimod IV arm. The difference in AEs between treatment arms was mainly driven by injection site reactions (ISRs). ISRs in the efgartigimod PH20 SC arm were all were mild (18/21 participants) or moderate (3/21 participants) in severity. Most ISRs were transient, resolving without treatment, and none led to treatment discontinuation. The most common ISR events (occurring in ≥5% of participants) included injection site rash, injection site erythema, injection site pruritis, injection site bruising, and injection site pain. In ADAPT-SC, the frequency of ISRs decreased with each injection of the treatment cycle (21.8%, first injection vs 10.2%, fourth injection), and in this interim analysis of ADAPT-SC+, with each treatment cycle (34.1%, cycle 1; 16.9%, cycle 2; 13.3%, cycle 3). Additionally, in ADAPT-SC+, participants who received efgartigimod IV in the antecedent studies have not demonstrated higher rates of ISRs compared with participants who received efgartigimod PH20 SC in the antecedent study. The next most common AE in both trials was headache; it occurred in 7 (12.7%) participants in each treatment arm of ADAPT-SC and in 25 (15.2%) of participants in this interim analysis of ADAPT-SC+. All were mild to moderate, with none leading to treatment discontinuation.

**Table 4:** Adverse event summary of ADAPT-SC and ADAPT-SC+ (safety analysis sets).

	ADAPT-SC (PD Noninferiority Study)						ADAPT-SC+ (Open-Label Extension Study)		
	EFG PH20 SC (n ​= ​55) [10.73 PY]			EFG IV (n ​= ​55) [10.53 PY]			EFG PH20 SC (n ​= ​164) [72.1 PY]		
	n (%)	No. events	ER^a^	n (%)	No. events	ER^a^	n (%)	No. events	ER^a^
**AEs**	37 (67.3)	133	12.4	28 (50.9)	80	7.6	125 (76.2)	790	11.0
**SAEs**	8 (14.5)	10	0.9	4 (7.3)	5	0.5	17 (10.4)	22	0.3
**AESIs (infections)**^b^	10 (18.2)	10	0.9	9 (16.4)	10	0.9	48 (29.3)	76	1.1
**Treatment discontinued due to AEs**	2 (3.6)	2	0.2	0 (0.0)	0	–	3 (1.8)	4	0.1
**Severe AEs (grade** ​≥**3)**	9 (16.4)	11	1.0	4 (7.3)	5	0.5	19 (11.6)	41	0.6
**Death**	0 (0.0)	0	–	0 (0.0)	0	–	2 (1.2)^c^	3	<0.1
**Most frequent AEs (occurring in >10% of participants), n (%)**									
Injection site reactions (localized)^d^	21 (38.2)	39	3.6	1 (1.8)^e^	0	–	69 (42.1)	307	4.3
Headache	7 (12.7)	10	0.9	7 (12.7)	11	1.0	25 (15.2)	58	0.8
COVID-19	2 (3.6)	2	0.2	0 (0.0)	0	–	19 (11.6)	20	0.3
Myasthenia gravis	6 (10.9)	8	0.7	1 (1.8)	2	0.2	7 (4.3)	10	0.1

AE, adverse event; AESI, adverse event of special interest; ER, event rate; IMP, investigational medicinal product; IV, intravenous; n, number of participants with at least 1 event; SAE, serious adverse event; SC, subcutaneous; SOC, system organ class; PD, pharmacodynamics; PY, participant-years.

Table 4 footnote: The safety analysis set included all randomly assigned participants who were exposed to the IMP.

a ER was calculated as number of events per participant-year of follow-up.

b AEs in the infections and infestations SOC were defined as AESIs because patients with MG are predisposed to infections, likely related to use of immunosuppressive treatment, and because efgartigimod causes a transient reduction in total IgG level.

c Causes of death were renal cancer metastatic in 1 participant and COVID-19/respiratory failure in 1 participant; neither event was considered by investigator to be related to IMP.

d Most common injection site reactions (≥5% of the participants in ADAPT-SC+) were erythema, pruritus, pain, bruising, rash, swelling.

e No preferred term AEs of injection-site reaction recorded. This AE was incorrectly coded (should have been catheter site reaction).

In the ADAPT-SC study, 8 (14.5%) participants in the efgartigimod PH20 SC arm had serious AEs (SAEs) compared with 4 (7.3%) in the efgartigimod IV arm. The most commonly reported SAE was MG worsening (5 in the efgartigimod PH20 SC and 1 in the efgartigimod IV arm); of these participants, all 6 achieved CMI in MG-ADL and 5 achieved CMI in QMG prior to the MG worsening event. Across both arms, all but 1 (which occurred on day 1 of treatment) of the SAEs of MG worsening were reported toward the end of the 7-week follow-up period. Five of the participants with SAEs of MG worsening continued treatment in ADAPT-SC+. In ADAPT-SC+, the incidence of MG worsening for this analysis was 4.3% (7/164 participants), including 1 incident of MG crisis; this event led to treatment discontinuation.

Infections are considered AEs of special interest (AESIs) because patients with MG are predisposed to infections, likely related to use of immunosuppressive treatment, and treatment with efgartigimod results in transient reductions in IgG level. In ADAPT-SC, infection was reported in 10 (18.2%) participants in the efgartigimod PH20 SC arm and 9 (16.4%) participants in the efgartigimod IV arm, with the majority being mild to moderate in severity. Most common were urinary tract infections (3.6% of participants), COVID-19 (1.8% of participants), and pharyngitis (1.8% of participants). There was only 1 serious infection (grade 3 cellulitis, not at injection site) and 1 non-serious infection (COVID-19, which resulted in treatment discontinuation), both in the efgartigimod PH20 SC arm. In this ADAPT-SC+ interim analysis, infections occurred in 48 of 164 (29.3%) participants. The majority were mild or moderate in severity; serious infections occurred in 6 of 164 (3.7%) participants reporting 10 events. These included COVID-19 (4), infectious diarrhea (1), peritonitis (1), sepsis (1), pneumonia (1), rotavirus infection (1), and localized infection of the left arm (1, not at injection site). No opportunistic infections were observed during ADAPT-SC or ADAPT-SC+ and none of the observed infections were reactivations of a previous infection.

The incidence in ADAPT-SC of ADAs against efgartigimod was 34.5% (19/55) in the PH20 SC arm and 20.0% (11/55) in the IV arm; of these, 12 had a positive ADA sample at baseline (7 in PH20 SC arm, 5 in IV arm). Incidence of NAbs against efgartigimod was 3.6% (2/55) in both arms, and incidence of antibodies against rHuPH20 was 5.5% (3/55) in the efgartigimod PH20 SC arm. NAbs against rHuPH20 were not detected. ADAs and NAbs against efgartigimod, as well as antibodies against rHuPH20, had no apparent impact on pharmacokinetics, PD, clinical efficacy, or safety parameters in ADAPT-SC or ADAPT-SC+ as of data cutoff.

Efgartigimod (in either PH20 SC or IV formulations) did not lead to reductions in serum albumin level nor to elevations in cholesterol. There were no clinically meaningful changes from baseline in vital signs, electrocardiograms, or physical examination findings in ADAPT-SC or ADAPT-SC+ thus far.

## Discussion

The ADAPT-SC phase 3 study demonstrated NI of efgartigimod PH20 SC 1000 ​mg to efgartigimod IV 10 ​mg/kg in percentage change from baseline in total IgG level at week 4. Onset and magnitude of reductions in total IgG level (and AChR-Ab level in AChR-Ab+ participants) aligned with maximal clinical improvements and were similar across treatment arms. Interim results from ADAPT-SC+ have demonstrated long-term safety and tolerability, as well as consistent and repeatable IgG reduction and efficacy across multiple treatment cycles with efgartigimod PH20 SC.

Efficacy in ADAPT-SC for efgartigimod PH20 SC and efgartigimod IV was consistent with results from the placebo-controlled ADAPT study, in which a broad population of participants with gMG demonstrated significant clinical improvements in both participant-reported and physician-assessed measures [10]. In ADAPT-SC, clinical improvement was seen as early as 1 week following treatment initiation, with maximal MG-ADL and QMG improvements at week 4 in both arms. The majority of participants in both treatment arms continued to have CMIs in MG-ADL and QMG at the end of the study (week 10), demonstrating extended clinical benefit from 1 cycle of treatment. Many participants experienced a robust magnitude of improvement, with 45.5% of AChR-Ab+ participants in the efgartigimod PH20 SC arm and 41.3% in the efgartigimod IV arm achieving MSE, similar to responses observed in the first cycle of the ADAPT study (40.0%, efgartigimod vs 11.1%, placebo) [10]. A consistent pattern of clinical improvement was demonstrated across multiple treatment cycles in ADAPT-SC+, with 33.3%–39.5% of AChR-Ab+ participants achieving MSE across cycles 1 through 3. Clinical efficacy was also seen in AChR-Ab– participants in ADAPT-SC and ADAPT-SC+, which adds to the growing body of evidence on efgartigimod efficacy in AChR-Ab– participants. These data are especially encouraging, considering the significant disease burden and unmet need experienced by patients with AChR-Ab– gMG [30].

In ADAPT-SC, efgartigimod PH20 SC and efgartigimod IV were both well tolerated. ISRs have been observed with use of subcutaneously administered biologics [31]; however, ISRs in the efgartigimod PH20 SC arm of ADAPT-SC and in participants in ADAPT-SC+ were all mild to moderate in severity, with none leading to treatment discontinuation. As of data cutoff in ADAPT-SC+, ISRs decreased with each treatment cycle. The rate of ISRs across both studies is within the reported range for other rHuPH20-containing SC products (7%–91%) [32]. In ADAPT-SC, serious adverse effects of MG worsening occurred in 6 patients; however, 5 of these events were reported toward the end of the 7-week follow-up period when retreatment was not allowed, and 5 of these participants continued treatment in ADAPT-SC+. One incident of MG crisis occurred in ADAPT-SC+, which is not uncommon in long-term MG studies [33], due to the fluctuating nature of MG symptoms.

Incidence of infections is higher in patients with gMG than in those without the disease, potentially predisposed by use of immunosuppressive treatment [34,35]. Importantly, while efgartigimod does reduce total IgG level, it does not lead to complete IgG removal and the reduction is transient, with no impact on other immunoglobulins. Preclinical studies of FcRn blockade have shown there is no impact on IgG production [36] and it does not prevent the generation of an immune response to vaccination [37]. Infection AEs occurred in 19 (17.3%) participants after 1 cycle of treatment in ADAPT-SC and 48 (29.3%) participants with up to 6 cycles of treatment in this interim analysis of ADAPT-SC+. Most infections were mild to moderate and did not lead to treatment discontinuation. Infection types and rates were consistent with those commonly reported in patients with gMG [34]. Lastly, efgartigimod (both PH20 SC and IV) neither decreased serum albumin nor increased cholesterol levels, both of which have been reported with other FcRn antagonists [11,38].

Results of the phase 3 ADAPT-SC study demonstrate NI of efgartigimod PH20 SC compared to efgartigimod IV in percentage change in total IgG level at week 4. Both formulations provided CMIs and were well tolerated. Interim analyses from the ongoing ADAPT-SC+ ​demonstrate long-term safety and tolerability, as well as repeatable clinical benefit across multiple treatment cycles. Collective data from ADAPT-SC and ADAPT-SC+ further demonstrate the utility of selective IgG reduction as a treatment approach for a broad population of patients with gMG. In conclusion, efgartigimod PH20 SC provides the potential for a broader treatment offering to the gMG community while meeting the needs of patients via an additional route of administration.

## Data availability statement

Argenx is committed to responsible data sharing regarding the clinical trials they fund. Included in this commitment is access to anonymized individual and trial-level data (analysis datasets) and other information (eg, protocols and clinical study reports), as long as the trials are not part of an ongoing or planned regulatory submission. This includes requests for clinical trial data for unlicensed products and indications. These clinical trial data can be requested by qualified researchers who engage in rigorous independent scientific research and will only be provided after review and approval of a research proposal and statistical analysis plan and execution of a data sharing agreement. Data requests can be submitted at any time and the data will be accessible for 12 months. Requests can be submitted to ESR@argenx.com.

## Author Contributions

All authors fulfilled ICMJE criteria for authorship. They had full access to study data; reviewed, edited, and provided final approval of the manuscript content; had final responsibility for the decision to submit for publication; and are accountable for the content of the work.

## Declaration of competing interest

This study was sponsored by argenx (Ghent, Belgium), the manufacturer of efgartigimod IV and efgartigimod PH20 SC. Efgartigimod IV has received regulatory approval for the treatment of gMG in multiple countries. Efgartigimod PH20 SC was approved for use in gMG by the US Food and Drug Administration. Medical writing and editorial support were funded by argenx. Li Liu, Fien Gistelinck, Sophie Steeland, Benjamin Van Hoorick, Jana Podhorna, and Filip Borgions are employees of argenx. Jan Noukens is partner at Curare Consulting and is a paid consultant for argenx. James F. Howard, Jr has received research support (paid to his institution) from Alexion Pharmaceuticals, argenx, Cartesian Therapeutics, the US Centers for Disease Control and Prevention, the Myasthenia Gravis Foundation of America, the Muscular Dystrophy Association, the US National Institutes of Health (including the National Institute of Neurological Disorders and Stroke and the National Institute of Arthritis and Musculoskeletal and Skin Diseases), Patient-Centered Outcomes Research Institute, Ra Pharmaceuticals (now UCB Biosciences), and Millennium Pharmaceuticals/Takeda Pharmaceuticals; honoraria from AcademicCME, Alexion Pharmaceuticals, argenx BV, Biologix Pharma, F. Hoffman-LaRoche Ltd, Horizon Therapeutics plc, Merck EMD Serono, NMD Pharma, Novartis Pharmaceuticals, PeerView CME, Ra Pharmaceuticals (now UCB Biosciences), Regeneron Pharmaceuticals, and Sanofi US; and non-financial support from Alexion Pharmaceuticals, argenx BV, Ra Pharmaceuticals (now UCB Biosciences), Toleranzia AB, and ZaiLab. Tuan Vu serves as site principal investigator for MG clinical trials sponsored by argenx, Alexion, UCB/Ra, Cartesian, Horizon/Viela Bio, Janssen/Momenta, Regeneron, Immunovant, and Sanofi and has served as consultant and/or speaker for argenx, Alexion, and UCB/Ra. George Li has nothing to disclose. Denis Korobko has received speaker honoraria from Roche-Moscow, Novartis Russia, Sanofi, Merck, Janssen (Johnson & Johnson company), BIOCAD; research grants from Novartis Russia, UCB, argenx, Viela Bio Inc. (now Horizon Therapeutics), Sanofi, Bristol Myers Squibb, Hoffman-LaRoche Ltd.; and has served on scientific advisory boards for Novartis Russia, Merck, Janssen (Johnson & Johnson company), and BIOCAD. Marek Smilowski has nothing to report. Yuebing Li has served on advisory boards for argenx, Catalyst, Immunovant, and UCB Pharma and has received grant support from argenx. Kimiaki Utsugisawa has served as a paid consultant for argenx, UCB Pharma, Janssen Pharma, Viela Bio, Chugai Pharma, Merck, and Mitsubishi Tanabe Pharma and has received speaker honoraria from argenx, Alexion Pharmaceuticals, UCB Pharma, and the Japan Blood Products Organization. Heinz Wiendl receives honoraria for acting as a member of scientific advisory boards for AbbVie, Alexion, argenx, Bristol Myers Squibb, Janssen, Merck, Novartis, and Sandoz; has received speaker honoraria and travel support from Alexion, Biogen, Bristol Myers Squibb, Genzyme, Merck, Neurodiem, Novartis, Ology, Roche, Teva, and WebMD Global; has received research funding from Deutsche Forschungsgesellschaft (DFG), Deutsche Myasthenie Gesellschaft e.V., European Union, Alexion, Amicus Therapeutics, argenx, Biogen, CSL Behring, F. Hoffmann-La Roche, Genzyme, Merck KgaA, Novartis, Roche Pharma, and UCB Biopharma; and is a paid consultant for AbbVie, Actelion, argenx, BD, Bristol Myers Squibb, EMD Serono, Fondazione Cariplo, Gossamer Bio, Idorsia, Immunic, Immunovant, INmune Bio, Syneos Health, Janssen, Merck, NexGen, Novartis, Roche, Sanofi, Swiss Multiple Sclerosis Society, UCB, and Worldwide Clinical Trials. Jan L. De Bleecker has served as a paid consultant for or received speaker honoraria from argenx, UCB Pharma, Alexion Pharmaceuticals, Sanofi, CSL Behring, and Roche. Renato Mantegazza has received funding for travel, meeting attendance, or advisory board participation from Alexion, argenx, Biomarin, Catalyst, Sanofi, Regeneron, and UCB.
