# Supplementary material for: Subcutaneous efgartigimod PH20 in generalized myasthenia gravis: A phase 3 randomized noninferiority study (ADAPT-SC) and interim analyses of a long-term open-label extension study (ADAPT-SC+)
Source: Neurotherapeutics. 2024 Sep 2;21(5):e00378. doi: 10.1016/j.neurot.2024.e00378 (PMC11579873; doi:10.1016/j.neurot.2024.e00378)
Supplement: Multimedia component 1 [file mmc1.docx]

**Supplemental Table 1. All Inclusion and Exclusion Criteria for ADAPT-SC and ADAPT-SC+**

| **Inclusion Criteria (ADAPT-SC)**  Participants are eligible to be included in the study only if *all* of the following criteria apply:   1. Must be capable of giving signed informed consent, which includes compliance with the requirements and restrictions listed in the informed consent form (ICF) and in protocol 2. At least 18 years of age at the time of signing the ICF 3. Diagnosed with gMG with confirmed documentation and supported by at least 1 of the following:    1. History of abnormal neuromuscular transmission demonstrated by single fiber electromyography or repetitive nerve stimulation    2. History of positive edrophonium test    3. Demonstrated improvement in MG signs upon treatment with oral acetylcholinesterase (AChE) inhibitors as assessed by the treating physician 4. Meeting the clinical criteria as defined by the Myasthenia Gravis Foundation of America (MGFA) class II, III, or IV 5. Abdominal skin tissue allows for absorption and assessment of local safety of the planned SC injection, as determined by the investigator 6. An MG-ADL total score of ≥5 points, with more than 50% of the score due to non-ocular symptoms at screening and baseline 7. Receiving a stable dose of other gMG treatment (concomitant gMG treatment) prior to screening. For patients receiving nonsteroidal immunosuppressants (NSISTs), steroids, and/or AChE inhibitors as concomitant medications, the following dose conditions apply:    1. NSISTs (e.g., azathioprine, methotrexate, cyclosporine, tacrolimus, mycophenolate mofetil, and cyclophosphamide): treatment initiated at least 6 months prior to screening and no changes to dose in the 3 months before screening    2. Steroids: treatment initiated at least 3 months prior to screening and no dose changes in the month before screening    3. AChE inhibitors: stable dose with no dose escalation during the 2 weeks before screening. AChE inhibitors must be withheld for at least 12 hours before the QMG assessment, to be consistent with the revised manual for the QMG test, as recommended by the MGFA 8. Contraceptive use should be consistent with local regulations regarding the methods of contraception for those participating in clinical studies and:    1. Male participants are eligible to participate if they agree to the following during the intervention period and for at least 90 days after the last study dose of the IMP:       1. Refrain from donating sperm    2. Plus, either       1. Be abstinent from heterosexual intercourse as their preferred and usual lifestyle (abstinent on a long term and persistent basis) and agree to remain abstinent    3. Or       1. Must agree to use a male condom with a female partner using an additionally highly effective contraceptive method with a failure rate of <1% per year when having sexual intercourse with a woman of childbearing potential who is not currently pregnant    4. Or       1. Be a sterilized man who has had a documented absence of sperm post-procedure    5. Female participants are eligible to participate if they are not pregnant or breastfeeding, and they are 1 of the following:       1. Women of nonchildbearing potential (WONCBP)    6. Or       1. Women of childbearing potential (WOCBP) and is using a contraceptive method that is highly effective (with a failure rate of <1% per year) during the study intervention and for at least 90 days after the last study dose of the IMP. The investigators should evaluate the potential for contraceptive method failure (e.g., noncompliance) in relationship to the first dose of the study intervention       2. WOCBP must have a negative highly sensitive serum pregnancy test within the screening period before the first dose of study IMP    7. Additional requirements for pregnancy testing during and after study intervention    8. The investigator is responsible for review of medical history, menstrual history, and recent sexual activity to decrease the risk for inclusion of a woman with an early undetected pregnancy | **Inclusion Criteria (ADAPT-SC+)**  Participants are eligible to be included in the study only if *all* of the following criteria apply:   1. Must be capable of giving signed informed consent, which includes compliance with the requirements and restrictions listed in the ICF and in protocol 2. Previously participated in antecedent studies ARGX-113-2001 or ARGX-113-1705 and are eligible for rollover as defined by:    1. For ARGX-113-2001       1. Completed the study and performed the EoS visit, or       2. Were discontinued from study treatment for reasons other than pregnancy or an (S)AE. Receiving rescue therapy is not exclusionary unless given in a response to a life-threatening situation    2. For ARGX-113-1705       1. Received the previous dose of efgartigimod IV at least 30 days prior to entry into this study, completed at least 1 year of study ARGX-113-1705, and performed the early discontinuation visit in ARGX-113-1705       2. Did not have 3 consecutive treatment failures in ARGX-113-1705, even if the participant received rescue therapy (unless rescue therapy was given in response to a life-threatening situation). Treatment failure is defined as the absence of a decrease of at least 2 points in total MG-ADL score compared to the subsequent treatment period baseline in at least 50% of the assessments       3. Are still receiving concomitant gMG medication. Participants who have stopped taking any concomitant medication for gMG are not eligible for rollover 3. Contraceptive use by men and women should be consistent with local regulations regarding the methods of contraception for those participating in clinical studies and:    1. Male participants are not allowed to donate sperm from signing the ICF until the end of the study.    2. Female participants – women of childbearing potential must have a negative urine pregnancy test at baseline before IMP can be administered |
| --- | --- |
| **Exclusion Criteria (ADAPT-SC)**  Participants are excluded from the study if *any* of the following criteria apply:   1. Are pregnant or lactating, or intend to become pregnant during the study or within 90 days after the last dose of IMP 2. Has any of the following medical conditions:    1. Clinically significant uncontrolled active or chronic bacterial, viral, or fungal infection at screening    2. Any other known autoimmune disease that, in the opinion of the investigator, would interfere with an accurate assessment of clinical symptoms of myasthenia gravis or put the participant at undue risk    3. History of malignancy unless deemed cured by adequate treatment with no evidence of reoccurrence for ≥3 years before the first administration of the IMP. Participants with the following cancers can be included at any time:       1. adequately treated basal cell or squamous cell skin cancer       2. carcinoma in situ of the cervix       3. carcinoma in situ of the breast       4. incidental histological findings of prostate cancer (TNM Classification of Malignant Tumors stage T1a or T1b)    4. Clinical evidence of other significant serious diseases, or the participant has had a recent major surgery, or who have any other condition that, in the opinion of the investigator, could confound the results of the study or put the participant at undue risk 3. Worsening muscle weakness secondary to concurrent infections or medications (aminoglycosides, beta-blockers, etc.) 4. A documented lack of clinical response to plasma exchange (PLEX) 5. Received a live-attenuated vaccine fewer than 28 days before screening. Receiving an inactivated subunit, polysaccharide, or conjugate vaccine any time before screening is not exclusionary 6. Received a thymectomy <3 months prior to screening or one is planned to be performed during the study period 7. The following results from these diagnostic assessments will be considered exclusionary:    1. Positive serum test at screening for an active viral infection with any of the following conditions:       1. Hepatitis B virus (HBV) that is indicative of an acute or chronic infection       2. Hepatitis C virus (HCV) based on HCV antibody assay       3. Human immunodeficiency virus (HIV) based on a CD4 count of ≤200 cells/mm^3^ or test results that are associated with an acquired immunodeficiency syndrome (AIDS)-defining condition, such as: Cytomegalovirus retinitis with loss of vision, *Pneumocystis jiroveci pneumonia*, chronic intestinal cryptosporidiosis, HIV-related encephalopathy, *Mycobacterium tuberculosis* (pulmonary or extrapulmonary), or invasive cervical cancer       4. Positive nasopharyngeal swab test for SARS-CoV-2 8. Using the following prior or concomitant therapies:    1. Use of an investigational product within 3 months or 5 half-lives (whichever is longer) before the first dose of IMP    2. Use of any monoclonal antibody within 6 months before the first dose of the IMP    3. Use of Ig administered intravenously (IVIg), SC (SCIg), or intramuscularly within 4 weeks of screening    4. Use of PLEX within 4 weeks of screening    5. Previously participated in a clinical study with efgartigimod and/or products coformulated with rHuPH20 and received at least 1 administration of IMP 9. Total IgG levels <6 g/L at screening 10. Current or history of (ie, within 12 months of screening) alcohol, drug, or medication abuse 11. A known hypersensitivity reaction to efgartigimod, rHuPH20, or any of its excipients | **Exclusion Criteria (ADAPT-SC+)**  Participants are excluded from the study if *any* of the following criteria apply:   1. The participant was discontinued early from studies ARGX-113-2001 or ARGX-113-1705, unless the reason for discontinuation from study ARGX-113-1705 was to roll over into study ARGX-113-2002    1. Participants who, in the investigator’s judgment, are not benefiting from efgartigimod IV in study ARGX-113-1705 Part B are not eligible for rollover into ARGX-113-2002 2. Are pregnant or lactating, or intend to become pregnant during the study or within 90 days after the last dose of IMP 3. Have any of the following medical conditions:    1. Clinically significant uncontrolled chronic bacterial, viral, or fungal infection at screening    2. Any other known autoimmune disease that, in the opinion of the investigator, would interfere with accurate assessment of clinical symptoms of myasthenia gravis or put the participant at undue risk    3. History of malignancy unless deemed cured by adequate treatment with no evidence of reoccurrence for ≥3 years before the first administration of IMP. Participants with the following cancers can be included at any time:       1. adequately treated basal cell or squamous cell skin cancer       2. carcinoma in situ of the cervix       3. carcinoma in situ of the breast       4. incidental histological findings of prostate cancer (TNM classification of malignant tumors stage T1a or T1b)    4. Clinical evidence of other significant serious diseases, or the participant has had a recent major surgery, or who have any other condition that, in the opinion of the investigator, could confound the results of the study or put the participant at undue risk 4. Received a live-attenuated vaccine within 28 days prior to study entry or plan to receive a live-attenuated vaccine during the study 5. Had a known hypersensitivity reaction to efgartigimod, rHuPH20, or any of its excipients |

AE, adverse event; EoS, end of study; gMG, generalized myasthenia gravis; ICF, informed consent form; IgG, immunoglobulin G; IMP, investigational medicinal product; MG, myasthenia gravis; MG-ADL, Myasthenia Gravis Activities of Daily Living; QMG, Quantitative Myasthenia Gravis; rHuPH20, recombinant human hyaluronidase PH20; SAE, serious adverse event; SARS-CoV-2, severe acute respiratory syndrome coronavirus 2; SC, subcutaneous; T, tumor; TNM, tumor, node, metastasis.
