# Supplementary material for: Subcutaneous efgartigimod PH20 in generalized myasthenia gravis: A phase 3 randomized noninferiority study (ADAPT-SC) and interim analyses of a long-term open-label extension study (ADAPT-SC+)
Source: Neurotherapeutics. 2024 Sep 2;21(5):e00378. doi: 10.1016/j.neurot.2024.e00378 (PMC11579873; doi:10.1016/j.neurot.2024.e00378)
Supplement: Multimedia component 2 [file mmc2.docx]

**Supplemental Table 2: Number and Percentage of AChR-Ab–Negative MG-ADL and QMG Responders in ADAPT-SC (ITT Population)**

|  | **EFG PH20 SC**  **n/N (%)** | **EFG IV**  **n/N (%)** | **Difference in response, %**  **(95% CI)** |
| --- | --- | --- | --- |
| **MG-ADL responders** |  |  |  |
| AChR-Ab–negative | 6/10 (60.0) | 5/9 (55.6) | 4.4 (−40.0 to 48.9) |
| **QMG responders** |  |  |  |
| AChR-Ab–negative | 5/10 (50.0) | 4/9 (44.4) | 5.6 (−39.3 to 50.4) |

AChR-Ab, acetylcholine receptor antibody; EFG, efgartigimod; ITT, intent to treat; IV, intravenous; MG-ADL, Myasthenia Gravis Activities of Daily Living; n/N, number of participants for whom the observation was reported/number of participants in the analysis set; QMG, Quantitative Myasthenia Gravis; SC, subcutaneous.

**Supplemental Table 2 footnote**: The 95% CI for the difference in the percentage of MG-ADL and QMG responders in the 2 treatment arms was determined based on a two-sample t-test using Satterthwaite’s correction. “MG-ADL responder” was defined as a participant with a reduction of ≥2 points from baseline for ≥4 consecutive weeks after onset, with onset of score reduction occurring, at the latest, 1 week after the last infusion or injection. “QMG responder” was defined as a participant with a reduction of ≥3 points from baseline for ≥4 consecutive weeks after onset, with onset of score reduction occurring, at the latest, 1 week after the last infusion or injection.
