# Supplementary material for: Subcutaneous efgartigimod PH20 in generalized myasthenia gravis: A phase 3 randomized noninferiority study (ADAPT-SC) and interim analyses of a long-term open-label extension study (ADAPT-SC+)
Source: Neurotherapeutics. 2024 Sep 2;21(5):e00378. doi: 10.1016/j.neurot.2024.e00378 (PMC11579873; doi:10.1016/j.neurot.2024.e00378)
Supplement: Multimedia component 3 [file mmc3.docx]

**Supplemental Table 3: ADAPT-SC and ADAPT-SC+ Study Groups**

| **ADAPT-SC Study Group** | | |
| --- | --- | --- |
| **Investigator** | **Site** | **Country** |
| Jan De Bleecker | AZ Sint-Lucas Gent, Department of Neurology | Belgium |
| Annelien De Pue | AZ Sint-Lucas Gent, Department of Neurology | Belgium |
| Temur Margania | Ltd New Hospitals, Department of Neurology | Georgia |
| Nino Totadze | Ltd New Hospitals, Department of Neurology | Georgia |
| Roman Shakarishvili | Ltd Petre Sarajishvili Institute of Neurology | Georgia |
| Elene Nabadze | Ltd Petre Sarajishvili Institute of Neurology | Georgia |
| Nana Kvirkvelia | Ltd Petre Sarajishvili Institute of Neurology | Georgia |
| Alexander Tsiskaridze | Pineo Medical Ecosystem Ltd | Georgia |
| Tamar Vashadze | Pineo Medical Ecosystem Ltd | Georgia |
| Lela Tavzarashvili | Pineo Medical Ecosystem Ltd | Georgia |
| Gvantsa Lomsadze | Pineo Medical Ecosystem Ltd | Georgia |
| Diana Sanikidze | Pineo Medical Ecosystem Ltd | Georgia |
| Gvantsa Giorgadze | Aversi Clinic Ltd | Georgia |
| Lili Chinchaladze | Aversi Clinic Ltd | Georgia |
| Nana Guldedava | Medi Club Georgia LLC | Georgia |
| Nino Akiashvili | Medi Club Georgia LLC | Georgia |
| Andreas Meisel | Charité Universitätsmedizin Berlin | Germany |
| Benjamin Hotter | Charité Universitätsmedizin Berlin | Germany |
| Sarah Hoffmann | Charité Universitätsmedizin Berlin | Germany |
| Heinz Wiendl | Klinik für Neurologie mit Institut für Translationale Neurologie | Germany |
| Jan Lunemann | Klinik für Neurologie mit Institut für Translationale Neurologie | Germany |
| Luisa Klotz | Klinik für Neurologie mit Institut für Translationale Neurologie | Germany |
| Marc Pawlitzki | Klinik für Neurologie mit Institut für Translationale Neurologie | Germany |
| Maria Judit Molnar | Semmelweis Egyetem, Genomikai Medicina és Ritka Betegsegek Intezete | Hungary |
| Agnes Palasti | Semmelweis Egyetem, Genomikai Medicina és Ritka Betegsegek Intezete | Hungary |
| Renato Mantegazza | Fondazione I.R.C.C.S. Istituto Neurologico Carlo Besta UOC Neurologia 4, Malattie Neuromuscolari | Italy |
| Annamaria Gallone | Fondazione I.R.C.C.S. Istituto Neurologico Carlo Besta UOC Neurologia 4, Malattie Neuromuscolari | Italy |
| Silvia Bonanno | Fondazione I.R.C.C.S. Istituto Neurologico Carlo Besta UOC Neurologia 4, Malattie Neuromuscolari | Italy |
| Carlo Antozzi | Fondazione I.R.C.C.S. Istituto Neurologico Carlo Besta UOC Neurologia 4, Malattie Neuromuscolari | Italy |
| Rita Frangiamore | Fondazione I.R.C.C.S. Istituto Neurologico Carlo Besta UOC Neurologia 4, Malattie Neuromuscolari | Italy |
| Fiametta Vanoli | Fondazione I.R.C.C.S. Istituto Neurologico Carlo Besta UOC Neurologia 4, Malattie Neuromuscolari | Italy |
| Lorenzo Maggi | Fondazione I.R.C.C.S. Istituto Neurologico Carlo Besta UOC Neurologia 4, Malattie Neuromuscolari | Italy |
| Andreetta Francesca | Fondazione I.R.C.C.S. Istituto Neurologico Carlo Besta UOC Neurologia 4, Malattie Neuromuscolari | Italy |
| Chiara Bossi | Fondazione I.R.C.C.S. Istituto Neurologico Carlo Besta UOC Neurologia 4, Malattie Neuromuscolari | Italy |
| Elena Rinaldi | Fondazione I.R.C.C.S. Istituto Neurologico Carlo Besta UOC Neurologia 4, Malattie Neuromuscolari | Italy |
| Elena Rousseau | Fondazione I.R.C.C.S. Istituto Neurologico Carlo Besta UOC Neurologia 4, Malattie Neuromuscolari | Italy |
| Giovanni Antonini | Azienda Ospedaliera Universitaria Sant’Andrea | Italy |
| Antonio Lauletta | Azienda Ospedaliera Universitaria Sant’Andrea | Italy |
| Girolamo Alfieri | Azienda Ospedaliera Universitaria Sant’Andrea | Italy |
| Laura Fionda | Azienda Ospedaliera Universitaria Sant’Andrea | Italy |
| Laura Tufano | Azienda Ospedaliera Universitaria Sant’Andrea | Italy |
| Matteo Garibaldi | Azienda Ospedaliera Universitaria Sant’Andrea | Italy |
| Stafania Morino | Azienda Ospedaliera Universitaria Sant’Andrea | Italy |
| Elena Rossini | Azienda Ospedaliera Universitaria Sant’Andrea | Italy |
| Luca Leonardi | Azienda Ospedaliera Universitaria Sant’Andrea | Italy |
| Rocco Costanzo | Azienda Ospedaliera Universitaria Sant’Andrea | Italy |
| Akiyuki Uzawa | Chiba University Hospital | Japan |
| Manato Yasuda | Chiba University Hospital | Japan |
| Yosuke Onishi | Chiba University Hospital | Japan |
| Hiroyuki Akamine | Chiba University Hospital | Japan |
| Kimiaki Utsugisawa | Hanamaki General Hospital | Japan |
| Yuriko Nagane | Hanamaki General Hospital | Japan |
| Yasushi Suzuki | National Hospital Organization Sendai Medical Center | Japan |
| Genya Watanabe | National Hospital Organization Sendai Medical Center | Japan |
| Kenichi Tsukita | National Hospital Organization Sendai Medical Center | Japan |
| Masanori Takahashi | Osaka University Hospital | Japan |
| Tatsusada Okuno | Osaka University Hospital | Japan |
| Tomoya Kubota | Osaka University Hospital | Japan |
| Masayuki Masuda | Tokyo Medical University Hospital | Japan |
| Eri Watanabe | Tokyo Medical University Hospital | Japan |
| Makiko Naito | Tokyo Medical University Hospital | Japan |
| Nobuhiro Ido | Tokyo Medical University Hospital | Japan |
| Naoya Minami | National Hospital Organization Hokkaido Medical Center | Japan |
| Itaru Amino | National Hospital Organization Hokkaido Medical Center | Japan |
| Masaaki Nino | National Hospital Organization Hokkaido Medical Center | Japan |
| Ryoji Nagunuma | National Hospital Organization Hokkaido Medical Center | Japan |
| Sachiko Akimoto | National Hospital Organization Hokkaido Medical Center | Japan |
| Yusei Miyazaki | National Hospital Organization Hokkaido Medical Center | Japan |
| Kiyokazu Kawabe | Toho University Omori Medical Center | Japan |
| Takehisa Hirayama | Toho University Omori Medical Center | Japan |
| Jan Verschuuren | Leiden University Medical Center | Netherlands |
| Annabel Ruiter | Leiden University Medical Center | Netherlands |
| Linda Remijn-Nelissen | Leiden University Medical Center | Netherlands |
| Jeanette Wigbers | Leiden University Medical Center | Netherlands |
| Jules Van Benthem | Leiden University Medical Center | Netherlands |
| Pieteke Van Weperen | Leiden University Medical Center | Netherlands |
| Martijn Tannemaat | Leiden University Medical Center | Netherlands |
| Malgorzata Bilinska | Uniwersyteckie Centrum Kliniczne | Poland |
| Marek Halas | Uniwersyteckie Centrum Kliniczne | Poland |
| Krysztof Kaminski | Uniwersyteckie Centrum Kliniczne | Poland |
| Andrzej Szczudlik | Centrum Neurologii Klinicznej | Poland |
| Grazyna Zwolinska | Centrum Neurologii Klinicznej | Poland |
| Marek Smilowski | Wielospecjalistyczna Poradnia Lekarska Synapsis | Poland |
| Lech Szczechowski | Wielospecjalistyczna Poradnia Lekarska Synapsis | Poland |
| Urszula Chyrchel-Paszkiewicz | Prywatny Gabinet Lekarski U. Chyrchel-Paszkiewicz | Poland |
| Jakub Barycki | Prywatny Gabinet Lekarski U. Chyrchel-Paszkiewicz | Poland |
| Magdalena Ogorek | Prywatny Gabinet Lekarski U. Chyrchel-Paszkiewicz | Poland |
| Anna Kostera-Pruszczyk | Samodzielny Publiczny Centralny Szpital Kliniczny | Poland |
| Aleksandra Golenia | Samodzielny Publiczny Centralny Szpital Kliniczny | Poland |
| Piotr Szczidlik | Samodzielny Publiczny Centralny Szpital Kliniczny | Poland |
| Krzysztof Banaszkiewicz | Krakowski Szpital Specjalistyczny im. Jana Pawla II | Poland |
| Agnieszka Kuluga | Krakowski Szpital Specjalistyczny im. Jana Pawla II | Poland |
| Michal Blaz | Krakowski Szpital Specjalistyczny im. Jana Pawla II | Poland |
| Monika Ostrowska | Krakowski Szpital Specjalistyczny im. Jana Pawla II | Poland |
| Michal Michalski | Krakowski Szpital Specjalistyczny im. Jana Pawla II | Poland |
| Nadezhda Malkova | State Budgetary Healthcare Institution of Novosibirsk Region “State Novosibirsk Regional Clinical Hospital” | Russia |
| Ilona Vergunova | State Budgetary Healthcare Institution of Novosibirsk Region “State Novosibirsk Regional Clinical Hospital” | Russia |
| Fatima Stuchevskaya | Medical Centre Reavita Med SPb | Russia |
| Anna Abramova | Medical Centre Reavita Med SPb | Russia |
| Victoria Kotova | Medical Centre Reavita Med SPb | Russia |
| Eugeny Granatov | Medical Centre Reavita Med SPb | Russia |
| Francisco Javier Rodriguez de Riviera | Hospital Universitario La Paz - PPDS | Spain |
| Mireya Fernandez-Fournier Fernandez | Hospital Universitario La Paz - PPDS | Spain |
| Rafael Jenaro Martinez | Hospital Universitario La Paz - PPDS | Spain |
| Maria Salvado-Figueras | Hospital Universitario Vall d’Hebron - PPDS | Spain |
| Raul Juntas Morales | Hospital Universitario Vall d’Hebron - PPDS | Spain |
| Daniel Sanchez Tejerina San Jose | Hospital Universitario Vall d’Hebron - PPDS | Spain |
| Elena Cortes Vicente | Hospital de La Santa Creu i Sant Pau | Spain |
| Rodrigo Avarez Velasco | Hospital de La Santa Creu i Sant Pau | Spain |
| David Reyes-Leiva | Hospital de La Santa Creu i Sant Pau | Spain |
| Ana Vesperinas-Castro | Hospital de La Santa Creu i Sant Pau | Spain |
| Maria Teresa Sevilla Mantecon | Hospital Universitari i Politecnic La Fe de Valencia | Spain |
| Marina Frasquet Carrera | Hospital Universitari i Politecnic La Fe de Valencia | Spain |
| James F. Howard, Jr | University of North Carolina at Chapel Hill | United States |
| Manisha Chopra | University of North Carolina at Chapel Hill | United States |
| Rebecca Traub | University of North Carolina at Chapel Hill | United States |
| Tuan Vu | University of South Florida | United States |
| Jerrica Farias | University of South Florida | United States |
| Niranja Suresh | University of South Florida | United States |
| Tulio Bertorini | Wesley Neurology Clinic, PC | United States |
| Yaohui Chai | Wesley Neurology Clinic, PC | United States |
| Amanda Huffman | Wesley Neurology Clinic, PC | United States |
| Cindy Benzel | Wesley Neurology Clinic, PC | United States |
| Ratna Bhavaraju-Sanka | University of Texas - San Antonio - Health Science Center - PPDS | United States |
| Carlayne Jackson | University of Texas - San Antonio - Health Science Center - PPDS | United States |
| Yuebing Li | Cleveland Clinic Ohio | United States |
| Benjamin Claytor | Cleveland Clinic Ohio | United States |
| Gregory Sahagian | The Neurology Center of Southern California | United States |
| Yasmin Camberos | The Neurology Center of Southern California | United States |
| Estela Soto | The Neurology Center of Southern California | United States |
| April Tenorio | The Neurology Center of Southern California | United States |
| Guadalupe Sanchez | The Neurology Center of Southern California | United States |
| Benjamin Frishberg | The Neurology Center of Southern California | United States |
| Tara Quesnell | The Neurology Center of Southern California | United States |
| Kinjal Madhav | The Neurology Center of Southern California | United States |
| John Heinen | The Neurology Center of Southern California | United States |
| Andrew Bierman | The Neurology Center of Southern California | United States |
| Yessar Hussain | Austin Neuromuscular Center | United States |
| Casey Kafena | Austin Neuromuscular Center | United States |
| Luisa Sansalone | Austin Neuromuscular Center | United States |
| Michael Chiodo | Austin Neuromuscular Center | United States |
| Mary Trunk | Austin Neuromuscular Center | United States |
| Stephanie Gonsoulin | Austin Neuromuscular Center | United States |
| Leticia Camcho | Austin Neuromuscular Center | United States |
| Emil Hussain | Austin Neuromuscular Center | United States |
| Jeffrey Guptill | Duke Early Phase Clinical Research Unit | United States |
| Natalia Laura Gonzalez | Duke Early Phase Clinical Research Unit | United States |
| Moboluwade D. Abe-Lathan | Duke Early Phase Clinical Research Unit | United States |
| Shruti M. Raja | Duke Early Phase Clinical Research Unit | United States |
| Ellen Tuck | Duke Early Phase Clinical Research Unit | United States |
| Brian Costell | Neurology Offices of South Florida | United States |
| Dargelis Chi | Neurology Offices of South Florida | United States |
| George Li | Cleveland Clinic Ohio | United States |
| Jana Gray | Cleveland Clinic Ohio | United States |
| Albert Onzo | Cleveland Clinic Ohio | United States |
| Laura Pugh | Cleveland Clinic Ohio | United States |
| Maria Vasconcelos | Cleveland Clinic Ohio | United States |
| David Klein | Cleveland Clinic Ohio | United States |
| Victoria Georgoulis | Cleveland Clinic Ohio | United States |
| Giang Chau | Cleveland Clinic Ohio | United States |
| Tomas Holmlund | Dent Neurologic Institute | United States |
| Luisa Rojas Estupinan | Dent Neurologic Institute | United States |
| Thao-Dyen Dang | Dent Neurologic Institute | United States |
| Michelle Rainka | Dent Neurologic Institute | United States |
| Traci Aladeen | Dent Neurologic Institute | United States |
| Amanda Jamison | Dent Neurologic Institute | United States |
| Erica Mezzasalma | Dent Neurologic Institute | United States |
| Daryn Slazyk | Dent Neurologic Institute | United States |
| Bennett H Myers | Dent Neurologic Institute | United States |
| Tanya Geist | Dent Neurologic Institute | United States |
| Katelyn McCormack | Dent Neurologic Institute | United States |
| Wendy Callen | Dent Neurologic Institute | United States |
| **ADAPT-SC+ Study Group** | | |
| **Investigator** | **Site** | **Country** |
| Jan De Bleecker | AZ Sint-Lucas Gent, Department of Neurology | Belgium |
| Annelien De Pue | AZ Sint-Lucas Gent, Department of Neurology | Belgium |
| Stanislav Vohanka | Neurologicka klinika, LF MU a FN Brno | Czech Republic |
| Tomas Horak | Neurologicka klinika, LF MU a FN Brno | Czech Republic |
| Viktoria Kokosova | Neurologicka klinika, LF MU a FN Brno | Czech Republic |
| Magda Horakova | Neurologicka klinika, LF MU a FN Brno | Czech Republic |
| Temur Margania | Department of Neurology, New Hospitals | Georgia |
| Dali Kankava | Department of Neurology, New Hospitals | Georgia |
| Khatuna Sichinava | Department of Neurology, New Hospitals | Georgia |
| Roman Shakarishvili | Ltd Petre Sarajishvili Institute of Neurology | Georgia |
| Elene Nebadze | Ltd Petre Sarajishvili Institute of Neurology | Georgia |
| Nana Kvirkvelia | Ltd Petre Sarajishvili Institute of Neurology | Georgia |
| Alexander Tsiskaridze | Pineo Medical Ecosystem Ltd | Georgia |
| Tamar Vashadze | Pineo Medical Ecosystem Ltd | Georgia |
| Lela Tavzarashvili | Pineo Medical Ecosystem Ltd | Georgia |
| Gvantsa Giorgadze | Aversi Clinic Ltd | Georgia |
| Maka Mania | Aversi Clinic Ltd | Georgia |
| Nana Guldeva | MediClubGeorgia Ltd | Georgia |
| Nino Akiashvili | MediClubGeorgia Ltd | Georgia |
| Andreas Meisel | Charité Universitätsmedizin Berlin | Germany |
| Meret Herdick | Charité Universitätsmedizin Berlin | Germany |
| Frauke Stascheit | Charité Universitätsmedizin Berlin | Germany |
| Maike Stein | Charité Universitätsmedizin Berlin | Germany |
| Paolo Doksani | Charité Universitätsmedizin Berlin | Germany |
| Heinz Wiendl | Universitätsklinikum Münster | Germany |
| Catharina Korsukewitz | Universitätsklinikum Münster | Germany |
| Luisa Klotz | Universitätsklinikum Münster | Germany |
| Csilla Rózsa | Jahn Ferenc Dél-Pesti Kórház és Rendelointézet | Hungary |
| David Bors | Jahn Ferenc Dél-Pesti Kórház és Rendelointézet | Hungary |
| Maria Judit Molnar | Semmelweis Egyetem, Genomikai Medicina és Ritka Betegsegek Intezete | Hungary |
| Agnes Palasti | Semmelweis Egyetem, Genomikai Medicina és Ritka Betegsegek Intezete | Hungary |
| Renato Mantegazza | Fondazione I.R.C.C.S. Istituto Neurologico Carlo Besta UOC Neurologia 4, Malattie Neuromuscolari | Italy |
| Rita Frangiamore | Fondazione I.R.C.C.S. Istituto Neurologico Carlo Besta UOC Neurologia 4, Malattie Neuromuscolari | Italy |
| Carlo Antozzi | Fondazione I.R.C.C.S. Istituto Neurologico Carlo Besta UOC Neurologia 4, Malattie Neuromuscolari | Italy |
| Lorenzo Maggi | Fondazione I.R.C.C.S. Istituto Neurologico Carlo Besta UOC Neurologia 4, Malattie Neuromuscolari | Italy |
| Silvia Bonanno | Fondazione I.R.C.C.S. Istituto Neurologico Carlo Besta UOC Neurologia 4, Malattie Neuromuscolari | Italy |
| Fiametta Vanoli | Fondazione I.R.C.C.S. Istituto Neurologico Carlo Besta UOC Neurologia 4, Malattie Neuromuscolari | Italy |
| Francesco Saccà | Azienda Ospedaliera Universitaria Federico II | Italy |
| Nunzia Cuomo | Azienda Ospedaliera Universitaria Federico II | Italy |
| Chiara Pane | Azienda Ospedaliera Universitaria Sant’Andrea | Italy |
| Angela Marsili | Azienda Ospedaliera Universitaria Sant’Andrea | Italy |
| Giorgia Puorro | Azienda Ospedaliera Universitaria Sant’Andrea | Italy |
| Akiyuki Uzawa | Chiba University Hospital | Japan |
| Manato Yasuda | Chiba University Hospital | Japan |
| Yosuke Onishi | Chiba University Hospital | Japan |
| Hiroyuki Akamine | Chiba University Hospital | Japan |
| Kimiaki Utsugisawa | Hanamaki General Hospital | Japan |
| Yuriko Nagane | Hanamaki General Hospital | Japan |
| Yasushi Suzuki | National Hospital Organization Sendai Medical Center | Japan |
| Genya Watanabe | National Hospital Organization Sendai Medical Center | Japan |
| Kenichi Tsukita | National Hospital Organization Sendai Medical Center | Japan |
| Masanori Takahashi | Osaka University Hospital | Japan |
| Tatsusada Okuno | Osaka University Hospital | Japan |
| Tomoya Kubota | Osaka University Hospital | Japan |
| Shin Hisahara | Sapporo Medical University Hospital | Japan |
| Minoru Yamada | Sapporo Medical University Hospital | Japan |
| Rei Mayanaga | Sapporo Medical University Hospital | Japan |
| Shuichiro Suzuki | Sapporo Medical University Hospital | Japan |
| Taro Saito | Sapporo Medical University Hospital | Japan |
| Kazuna Ikeda | Sapporo Medical University Hospital | Japan |
| Masaki Saito | Sapporo Medical University Hospital | Japan |
| Naotoshi Iwahara | Sapporo Medical University Hospital | Japan |
| Masayuki Masuda | Tokyo Medical University Hospital | Japan |
| Eri Watanabe | Tokyo Medical University Hospital | Japan |
| Makiko Naito | Tokyo Medical University Hospital | Japan |
| Nobuhiro Ido | Tokyo Medical University Hospital | Japan |
| Naoya Minami | National Hospital Organization Hokkaido Medical Center | Japan |
| Itaru Amino | National Hospital Organization Hokkaido Medical Center | Japan |
| Masaaki Nino | National Hospital Organization Hokkaido Medical Center | Japan |
| Ryoji Nagunuma | National Hospital Organization Hokkaido Medical Center | Japan |
| Sachiko Akimoto | National Hospital Organization Hokkaido Medical Center | Japan |
| Yusei Miyazaki | National Hospital Organization Hokkaido Medical Center | Japan |
| Kiyokazu Kawabe | Toho University Omori Medical Center | Japan |
| Takehisa Hirayama | Toho University Omori Medical Center | Japan |
| Masaru Yanagihashi | Toho University Omori Medical Center | Japan |
| Jan Verschuuren | Leiden University Medical Center | Netherlands |
| Annabel Maria Ruiter | Leiden University Medical Center | Netherlands |
| Linda Remijn-Nelissen | Leiden University Medical Center | Netherlands |
| Martijn Tannemaat | Leiden University Medical Center | Netherlands |
| Malgorzata Bilinska | Uniwersyteckie Centrum Kliniczne | Poland |
| Marek Halas | Uniwersyteckie Centrum Kliniczne | Poland |
| Andrzej Szczudlik | Centrum Neurologii Klinicznej | Poland |
| Grazyna Zwolinska | Centrum Neurologii Klinicznej | Poland |
| Dorota Zawislak | Centrum Neurologii Klinicznej | Poland |
| Marek Smilowski | Wielospecjalistyczna Poradnia Lekarska Synapsis | Poland |
| Lech Szczechowski | Wielospecjalistyczna Poradnia Lekarska Synapsis | Poland |
| Urszula Chyrchel-Paszkiewicz | Prywatny Gabinet Lekarski U. Chyrchel-Paszkiewicz | Poland |
| Magdalena Ogorek | Prywatny Gabinet Lekarski U. Chyrchel-Paszkiewicz | Poland |
| Anna Kostera-Pruszczyk | Uniwersyteckie Centrum Kliniczne WUM, Centralny Szpital Kliniczny | Poland |
| Aleksandra Golenia | Uniwersyteckie Centrum Kliniczne WUM, Centralny Szpital Kliniczny | Poland |
| Piotr Szczidlik | Uniwersyteckie Centrum Kliniczne WUM, Centralny Szpital Kliniczny | Poland |
| Ewa Sobieszczuk | Uniwersyteckie Centrum Kliniczne WUM, Centralny Szpital Kliniczny | Poland |
| Agnieszka Kuluga | Krakowski Szpital Specjalistyczny im. Jana Pawla II | Poland |
| Michal Blaz | Krakowski Szpital Specjalistyczny im. Jana Pawla II | Poland |
| Monika Ostrowska | Krakowski Szpital Specjalistyczny im. Jana Pawla II | Poland |
| Michal Michalski | Krakowski Szpital Specjalistyczny im. Jana Pawla II | Poland |
| Krystian Majkowski | Krakowski Szpital Specjalistyczny im. Jana Pawla II | Poland |
| Anna Kurowska | Krakowski Szpital Specjalistyczny im. Jana Pawla II | Poland |
| Kacper Szewczyk | Krakowski Szpital Specjalistyczny im. Jana Pawla II | Poland |
| Denis Korobko | Novosibirsk State Regional Clinical Hospital | Russia |
| Anna Prokaeva | Novosibirsk State Regional Clinical Hospital | Russia |
| Ilona Vergunova | Novosibirsk State Regional Clinical Hospital | Russia |
| Ekaterina Tretyakova | Novosibirsk State Regional Clinical Hospital | Russia |
| Fatima Stuchevskaya | LLC “Medical Center” Reavita Med SPb | Russia |
| Raul Juntas Morales | Hospital Universitario Vall d’Hebron - PPDS | Spain |
| Javier Joaquin Sotoca Fernandez | Hospital Universitario Vall d’Hebron - PPDS | Spain |
| Veronica Lopez Diego | Hospital Universitario Vall d’Hebron - PPDS | Spain |
| Daniel Sanchez-Tejerina San Jose | Hospital Universitario Vall d’Hebron - PPDS | Spain |
| Maria Salvado-Figueras | Hospital Universitario Vall d’Hebron - PPDS | Spain |
| Arnau Llaurado | Hospital Universitario Vall d’Hebron - PPDS | Spain |
| Elena Cortez Vicente | Hospital de La Santa Creu i Sant Pau | Spain |
| David Reyes Leiva | Hospital de La Santa Creu i Sant Pau | Spain |
| Maria Teresa Sevilla Mantecon | Hospital Universitari i Politecnic La Fe de Valencia | Spain |
| Francisco Javier Cabello Murgui | Hospital Universitari i Politecnic La Fe de Valencia | Spain |
| Jesus Jimenez | Hospital Universitari i Politecnic La Fe de Valencia | Spain |
| Rafael Sevila Mantecon | Hospital Universitari i Politecnic La Fe de Valencia | Spain |
| Juan Sebastian Aller | Hospital Universitari i Politecnic La Fe de Valencia | Spain |
| Javier Caballero Daroqui | Hospital Universitari i Politecnic La Fe de Valencia | Spain |
| James F. Howard, Jr | University of North Carolina at Chapel Hill | United States |
| Manisha Chopra | University of North Carolina at Chapel Hill | United States |
| Rebecca Traub | University of North Carolina at Chapel Hill | United States |
| Tuan Vu | University of South Florida | United States |
| Jerrica Farias | University of South Florida | United States |
| Niranja Suresh | University of South Florida | United States |
| Kathleen Murray | University of South Florida | United States |
| Tulio Bertorini | Wesley Neurology Clinic, PC |  |
| Ratna Bhavaraju-Sanka | University of Texas - San Antonio - Health Science Center - PPDS | United States |
| Carlayne Jackson | University of Texas - San Antonio - Health Science Center - PPDS | United States |
| Yogeet Kaur | University of Texas - San Antonio - Health Science Center - PPDS | United States |
| Mamatha Pasnoor | University of Kansas Medical Center Research Institute | United States |
| Duaa Jabari | University of Kansas Medical Center Research Institute | United States |
| Mazen Dimachkie | University of Kansas Medical Center Research Institute | United States |
| Omar Jawdat | University of Kansas Medical Center Research Institute | United States |
| Constantine Farmakidis | University of Kansas Medical Center Research Institute | United States |
| Jeffrey Statland | University of Kansas Medical Center Research Institute | United States |
| Yuebing Li | Cleveland Clinic Ohio | United States |
| Benjamin Claytor | Cleveland Clinic Ohio | United States |
| Debbie Hastings | Cleveland Clinic Ohio | United States |
| Srikanth Muppidi | Stanford Neuroscience Health Center | United States |
| Yuen So | Stanford Neuroscience Health Center | United States |
| Neelam Goyal | Stanford Neuroscience Health Center | United States |
| Gregory Sahagian | North County Neurology Associates | United States |
| Benjamin Frishberg | North County Neurology Associates | United States |
| Tara Quesnell | North County Neurology Associates | United States |
| Kinjal Madhav | North County Neurology Associates | United States |
| John Heinen | North County Neurology Associates | United States |
| Vern Juel | Duke Early Phase Clinical Research Unit | United States |
| Natalia Gonzales | Duke Early Phase Clinical Research Unit | United States |
| Shruti Raja | Duke Early Phase Clinical Research Unit | United States |
| Brian Costell | Neurology Offices of South Florida | United States |
| Dargelis Chi | Neurology Offices of South Florida | United States |
| George Li | Medsol Clinical Research Center Inc | United States |
| David Klein | Medsol Clinical Research Center Inc | United States |
| Luisa Rojas Estupinan | Dent Neurologic Institute | United States |
| Wendy Callen | Dent Neurologic Institute | United States |
| Katelyn McCormack | Dent Neurologic Institute | United States |
| Traci Aladeen | Dent Neurologic Institute | United States |
| Michelle Rainka | Dent Neurologic Institute | United States |
| Bennett H. Myers | Dent Neurologic Institute | United States |
| Tanya Geist | Dent Neurologic Institute | United States |
